# PYK2 promotes cell proliferation and epithelial-mesenchymal transition in endometriosis by phosphorylating Snail1

**DOI:** 10.1186/s10020-025-01218-1

**Published:** 2025-04-27

**Authors:** Lu Liu, Lan Liu, Chenjing Yue, Shiyu Du, Jiayu Liu, Zhenhai Yu

**Affiliations:** 1 Department of Reproductive Medicine, Affiliated Hospital of Shandong Second Medical University, Weifang, Shandong Province P. R. China; 2 School of Clinical Medicine, Shandong Second Medical University, Weifang, Shandong Province P. R. China

**Keywords:** PYK2, Snail1, EMT, Endometriosis, VS-6063

## Abstract

**Background:**

Endometriosis can lead to decreased endometrial receptivity, reduced rates of implantation, and diminished ovarian reserve. Currently, more than 50% of infertile women are found to suffer from endometriosis. However the etiology and pathogenesis of endometriosis are still poorly understood. Epithelial-mesenchymal transition (EMT) has been confirmed to be involved in endometriosis. PYK2 is a non-receptor tyrosine kinase that affects cell proliferation, survival, and migration by regulating intracellular signaling pathways. PYK2 plays a regulatory role in the EMT process by affecting the expression of genes associated with EMT through the influence of transcription factors. Snail1 (Snail1) plays a key role in the EMT process and is highly expressed in endometriosis tissues. On the other hand, Snail1 affects the invasive and metastatic ability of endometriosis cells mainly by regulating the EMT process. However, the upstream mechanisms that regulate the process of Snail1 protein stability in endometriosis are not clear.

**Methods:**

We identified a non-receptor tyrosine kinase, proline-rich tyrosine kinase 2 (PYK2 or PTK2B), and examined the expression of PYK2 in endometriosis. The relevant plasmids were constructed. This study enrolled 20 patients with laparoscopically confirmed endometriosis meeting ASRM diagnostic criteria, collecting ectopic lesions (14 ovarian endometriotic cysts and 6 deep infiltrating nodules) along with matched eutopic endometrial tissues (15 proliferative phase, 5 secretory phase) as controls. All tissue specimens underwent immunohistochemical analysis. Human endometrial stromal cells (HESC) were isolated from normal endometrium of 3 control patients for in vitro meconium induction. Ectopic endometrial stromal cells (EESC) were obtained from 5 ectopic lesions. Protein extracts from both ectopic tissues and cells were subjected to Western blot and co-immunoprecipitation (Co-IP) interaction validation. Functional assays (proliferation/migration/invasion) were performed using EESC and 11Z cell lines with triplicate biological replicates. Co-IP experiments were performed to verify the interaction between PYK2 and Snail1, as well as to determine the specific location of this interaction. Additionally, we examined the effect of PYK2 on endometriosis cells in vitro and whether VS-6063 inhibits the biological functions of endometriosis cells. Endometriosis models were established in 20 five-week-old female C57BL/6 mice, randomly allocated into experimental (*n* = 10) and control (*n* = 10) groups. Statistical analyses were conducted using GraphPad Prism 7.0, employing parametric tests for normally distributed data and non-parametric methods otherwise, with Benjamini-Hochberg correction for multiple comparisons.

**Results:**

PYK2 is highly expressed in endometriosis tissues. It acts as a new binding partner of Snail1 and enhances EMT in endometriosis by increasing the phosphorylation of Snail1. Additionally, PYK2 promotes the proliferation, migration, and invasion of endometriosis cells while inhibiting decidualization. We demonstrated that VS-6063 inhibited the proliferation, migration, and invasion of endometriosis cells in vitro, as well as the growth of endometriotic lesions in vivo.

**Conclusions:**

PYK2 is a novel binding partner of Snail1. PYK2 promotes the occurrence and development of endometriosis by up-regulating Snail1, which could be a promising therapeutic target for endometriosis.

**Supplementary Information:**

The online version contains supplementary material available at 10.1186/s10020-025-01218-1.

## Background

Endometriosis is a prevalent disease that can cause infertility in women of childbearing age, impacting approximately 190 million fertile women globally (Edgley et al. 2023). Up to half of these infertile women are found to have endometriosis (Horne and Missmer 2022). Symptoms of endometriosis include chronic pain in the pelvic area and dyspareunia, can negatively impact physical and psychological health, quality of life, and productivity at work (Nnoaham et al. 2011). The causes of infertility in female patients with endometriosis include anatomical changes due to adhesions and fibrosis, endocrine abnormalities, and immune system disorders (Saunders and Horne 2021). Additionally, reduced endometrial receptivity, implantation rate, and ovarian reserve due to endometriosis also contribute to infertility (Pirtea et al. 2023). To date, the pathogenesis of endometriosis remains unclear, and there is no particularly significant diagnostic biomarker. All theories and studies on the etiology of endometriosis tend to focus on complex dysregulated hormonal signaling (Murphy et al. 2022; Bonavina and Taylor 2022). Another challenge lies in diagnosing endometriosis, as laparoscopy is no longer considered the gold standard according to the 2022 ESHRE endometriosis guidelines (Becker et al. 2022). Furthermore, delayed diagnosis of endometriosis is prevalent, with an average time span of 6–7 years from the onset of clinical symptoms to receiving a diagnosis (Nnoaham et al. 2011). Early detection and treatment are crucial for women experiencing infertility due to endometriosis. Treatment options for this condition encompass surgical excision of lesions and hormonal therapy. Nevertheless, surgical intervention may result in chronic post-surgical pain (CPSP) or the development of endometriosis in alternative locations like abdominal wall endometriosis (AWE) (Singh et al. 2020). Hormone therapy may alleviate pain symptoms temporarily, but it does not address the underlying issue of endometriosis. The problem of pain recurrence is common once the medication is discontinued, and infertility associated with endometriosis remains unaffected by hormone therapy (Koga et al. 2015). Therefore, it is essential to understand the detailed molecular mechanisms of endometriosis in order to generate new ideas for targeted therapies and achieve clinical remission in infertile women.

Despite being a benign disease, endometriosis exhibits clinical and biological behaviors similar to those of a malignant tumor, such as implantation, invasion, and metastasis (Marquardt et al. 2023). The involvement of EMT in the pathogenesis of endometriosis has been demonstrated by many studies over the past few years (Konrad et al. 2020). During the process of EMT, epithelial cells lose their polarity, leading to enhanced migration and invasion abilities. However, there is also an increase in extracellular matrix (ECM) degradation (Yao et al. 2011). Numerous studies have shown that EMT is a central developmental process in chronic inflammation, tumor metastasis, and other diseases (Yao et al. 2011). It is well known that Snail1 plays a pivotal role in inducing EMT, and elevated levels of Snail1 are associated with higher tumor grade and shorter patient survival times (Kaufhold and Bonavida 2014; Wang et al. 2013). Snail1, as a transcription factor, has been shown to be highly expressed in ectopic endometrial tissue, particularly in ovarian endometriosis (chocolate cyst of the ovary) (Xiong et al. 2019; Cai et al. 2018); however, the mechanism controlling the stability of the Snail1 protein remains unclear.

PYK2 belongs to the family of tyrosine kinases, just like focal adhesion kinase (FAK) and Src (Shen and Guo 2018; Franco and Tamagnone 2008). Tyrosine phosphorylation is a crucial post-translational protein modification that regulates intracellular signal transduction and responds to extracellular signals (Franco and Tamagnone 2008). Src plays a crucial role in several events related to tumor progression, and inhibitors that target Src are also considered promising drugs for cancer treatment (Guarino 2010). PYK2 plays a crucial role in promoting the aggressive spread and metastasis of cancer cells, as it coordinates adhesion and cytoskeletal dynamics to regulate cell migration, proliferation, and survival through signaling pathways (Lipinski and Loftus 2010). As a result, PYK2 is widely recognized as an oncogene and a potential target for targeted cancer therapies; however, its role in the development of endometriosis remains unknown.

In this study, we investigated the role of PYK2 in the development of endometriosis. We identified PYK2 as a novel binding partner of Snail1 and conducted comparative analysis. Our results revealed that ectopic endometrial tissue exhibited significantly increased expression of PYK2 compared to normal endometrial tissue. This upregulated expression of PYK2 plays a crucial role in promoting cell proliferation, migration, and invasion in endometriosis cells, suggesting a pivotal function of PYK2 in the pathogenesis of endometriosis. Additionally, PYK2 can directly phosphorylate Snail1, further promoting the progression of endometriosis. The PYK2 inhibitor VS-6063 inhibited the growth of endometriosis lesions in a mouse model. Overall, this study provides a theoretical basis for targeting PYK2 as a potential therapeutic option for endometriosis, which could potentially benefit infertile women with endometriosis in the future.

## Materials and methods

### Tissue collection

There were two groups in the study, each with 20 tissue samples. This study enrolled 20 patients with laparoscopically confirmed endometriosis meeting ASRM diagnostic criteria, collecting ectopic lesions (14 ovarian endometriotic cysts and 6 deep infiltrating nodules) along with matched eutopic endometrial tissues (15 proliferative phase, 5 secretory phase) as controls. According to the corresponding inclusion criteria (Table S1), patients from the Affiliated Hospital of Shandong Second Medical University were selected as the subjects for this experiment. The characteristics of the recruited subjects are shown in table S2. All tissues were obtained from the remaining specimens of clinical diagnosis and treatment and were used for the study with the approval of the Ethics Committee and the written informed consent of the patients, in accordance with the requirements of the Measures for Ethical Review of Biomedical Research Involving Human Beings. This study was approved by the Ethics Committee of the Affiliated Hospital of Shandong Second Medical University (wyfy-2022-ky-191).

### Immunohistochemistry (IHC)

The tissue blocks were fixed in 4% paraformaldehyde, embedded in paraffin, dried, and sectioned. The sections were deparaffinized in xylene and subsequently hydrated in ethanol. The hydrated sections were antigen-repaired, blocked with H_2_O_2_, and incubated with primary antibodies overnight. The corresponding secondary antibody was incubated the next day. After staining with DAB, the nucleus was counterstained with hematoxylin. Finally, the sections were dehydrated using ethanol, made transparent with xylene, and sealed with neutral gum. The antibodies used for IHC were as follows: Rabbit anti-PYK2 (Proteintech, Cat#17592-1-AP, 1:200), Mouse anti-PYK2 (Cell Signaling Technology, 3480 S, 1:200), Rabbit anti-Snail1 (Proteintech, Cat#13099-1-AP,1:200), Mouse anti-Snail1 (Cell Signaling Technology, #3895S, 1:250), Rabbit anti-Vimentin (Proteintech, Cat#10366-1-AP, 1:200) Rabbit anti-E-cadherin (Proteintech, Cat#20874-1-AP, 1:250), Rabbit anti-α-SMA (Proteintech, Cat#14395-1-AP, 1:200), Rabbit anti-β-catenin (Proteintech, Cat#51067-2-AP, 1:250), Rabbit anti-Ki67 (Proteintech, Cat#27309-1-AP, 1:4000) The immunohistochemical results were scored based on both the intensity of staining and the proportion of cells showing a definite positive reaction. The intensity of staining was assigned a score of 0 for negative staining, 1 for weak staining, 2 for moderate staining, and 3 for strong positive staining. The percentage of positive cells was classified as follows: 0 for 0–5%, 1 for 6–25%, 2 for 26–50%, 3 for 51–75%, and 4 for 76–100%. There were four categories based on staining grade: absent (0), weak (Edgley et al. 2023; Horne and Missmer 2022; Nnoaham et al. 2011), moderate (Saunders and Horne 2021; Pirtea et al. 2023; Murphy et al. 2022; Bonavina and Taylor 2022; Becker et al. 2022) or strong (Singh et al. 2020; Koga et al. 2015; Marquardt et al. 2023; Konrad et al. 2020).

### Cell culture and in vitro decidualization

The endometrial epithelium (11Z) cell line was established by Professor Anna Strazinski-Powitz (Gaetje et al. 1997; Zeitvogel et al. 2001; Romano et al. 2020). Human endometrial stromal cells (HESC) were isolated from normal endometrium of 3 control patients for in vitro meconium induction. The criteria for normal endometrial tissue were no endometrial polyps or polypoid hyperplasia, no atypical hyperplasia or endometrial cancer, and a postoperative pathological diagnosis of secretory phase endometrium. EESC cells were derived from five samples of endometriosis lesions, specifically cystic wall tissue from those patients with endometriosis cysts diagnosed by ultrasound or laparoscopy, which were collected mainly during the proliferative phase of the menstrual cycle. These endometriosis focal tissues were obtained from patients who underwent surgical intervention after ultrasound or laparoscopic diagnosis and the histopathologic diagnosis was confirmed by postoperative pathology. The collected tissues were cut into pieces measuring 1 mm³ using surgical scissors. 2 g of minced-tissue were transferred to cell dishes and incubated overnight at 37℃ after adding type IV collagenase (2 mg/mL), hyaluronidase (2 mg/mL), penicillin (100 U/mL) and streptomycin (100 µg/mL) and medium containing 20% fetal bovine serum. Impurities were removed using a 100-micron filter, and epithelial cells were removed through a 40-micron filter. The filtered mixture was then centrifuged, and the resulting precipitate was mixed with the culture medium. The mixture was placed in a cell incubator, and the culture medium was replaced after the cells adhered. The entire process was conducted under sterile conditions. Immunofluorescence staining for vimentin (Huang et al. 2020) and cytokeratin 7 (Le et al. 2017) confirmed the presence of EESC, HESC cells, and 11Z cells, as demonstrated in Supplementary Fig. S1A and Fig. S1B. The HEK293T cells were purchased from the Chinese Academy of Sciences. All cell lines were cultured in a 37℃-cell incubator with 5% CO_2_. The 11Z, HESC, and EESC cells were cultured in F12/DMEM liquid medium (Corning) containing 10% fetal bovine serum (Corning), while the HEK293T cells were cultured using DMEM medium. All cell lines have been passaged fewer than 15 times. The HESC cells were decidualized in vitro by incubating them with 10 nmol/L of estradiol-17β, 1µmol/L of progesterone and 0.5mmol/L of cAMP for 6 days. The medium was replaced every 48 h (Wang et al. 2018). Successful in vitro decidualization induces HESC to adopt a rounded morphology with expanded cytoplasm and indistinct cell borders—key features of decidualization cells.

### Cell proliferation assay

The cells that were transfected with the corresponding plasmids were re-plated into 24-well plates, and their cell numbers were counted every 24 h for a duration of 4 days. A statistical graph was created.

### Colony formation assay

The corresponding cells were seeded into the 6-well plate at a density of 400 cells per well. After incubating for 10–14 days, the medium was removed, and the cells were fixed with 4% paraformaldehyde for 15 min. Subsequently, they were stained with a crystal violet solution for 20 min. Finally, we captured photographs.

### Scratch wound-healing assay

The cells were seeded into 6-well plates and cultured until reached 100% cell density. Subsequently, the cells were then scratched with a medium pipette tip, washed three times with PBS, and photographed. Repeat the photograph again after 24 h.

### Transwell migration assay and matrigel invasion assay

The Transwell chamber with a pore diameter of 8 mm was prepared. Two hundred microliters of DMEM/F12 medium, containing approximately 80,000 cells, were added to the upper chamber, while 600µL of DMEM/F12 medium containing 10% fetal bovine serum was used as the liquid in the lower chamber. BD Biocoat Matrigel was mixed with DMEM/F12 medium (1:8) for Matrigel invasion assays. After incubating for 24 h at 37℃, the upper chamber was cleaned, fixed with 4% paraformaldehyde for 30 min, stained with a crystal violet hydrate solution for 20 min, and then photographed.

### Plasmids construction, antibodies and reagents

Plasmids construction was carried out as described previously (Lu et al. 2021; Han et al. 2022). pLVX-shRNA1 was constructed using short-hairpin RNA vectors and the relevant mutant plasmids were constructed using overlapping PCR techniques. Stable cell pools were constructed as previously described (Han et al. 2022). These products were sequenced without unexpected changes to the wild-type amino acid sequence. The primer sequences used are shown in Table S2. Information on relevant plasmids and antibodies is provided in the Supplementary Material (Table S3). The indicated cells were transfected using Lipofectamine 2000 according to the manufacturer’s instructions.

### Western blot

The corresponding cells were transfected with different plasmids. The collected samples were placed on ice, lysed with protein lysis buffer, centrifuged at 12,000 rpm for 10 min, and then the supernatant was mixed with SDS-PAGE loading buffer and incubated at 100 °C for 10 min. Quantifying protein concentrations standardizes them. The proteins were separated by SDS-PAGE, transferred to PVDF membranes using Bio-rad Criterion, and then incubated with the specified antibodies. The results were visualized using the Odyssey instrument. The antibodies used for Western Blot were as follows: Rabbit anti-PYK2 (Proteintech, Cat#17592-1-AP, 1:1500), Mouse anti-PYK2 (Cell Signaling Technology, 3480 S, 1:1500), Rabbit anti-Snail1 (Proteintech, Cat#13099-1-AP, 1:2000), Mouse anti-Snail1 (Cell Signaling Technology, #3895S, 1:2000), Rabbit anti-Vimentin (Proteintech, Cat#10366-1-AP, 1:2000), Rabbit anti-E-cadherin (Proteintech, Cat#20874-1-AP, 1:2500), Rabbit anti-α-SMA (Proteintech, Cat#14395-1-AP, 1:2000), Rabbit anti-β-catenin (Proteintech, Cat#51067-2-AP, 1:2500), Mouse anti-HA (Sigma-Aldrich, Cat#H3663, 1:3000), Mouse anti-Flag (Sigma-Aldrich, Cat#F1804, 1:2000), Mouse anti-β-actin (Sigma-Aldrich, Cat#A1978, 1:3000), Rabbit anti-HA (Proteintech, Cat#51064-2-AP, 1:2000), Rabbit anti-Flag (Proteintech, Cat#20543-1-AP, 1:3000), Rabbit anti-β-actin (Proteintech, Cat#20536-1-AP, 1:2000), Mouse anti-GFP (Sigma-Aldrich, Cat#G6539, 1:2000), Rabbit anti-PRL (Proteintech, Cat#16525-1-AP, 1:2000), Rabbit anti-IGFBP1, (Proteintech, Cat#13981-1-AP, 1:2500).

### Immunoprecipitation (IP)

The cells (5–8 × 10^5^)were treated with IP lysis buffer (Han et al. 2022). A portion of the supernatant was used as input. The remaining supernatant was incubated overnight at 4 °C after adding Protein A/G agarose beads. The beads were washed with Co-IP buffer more than five times and then subjected to immunoblotting (Ling et al. 2022; Lu et al. 2023). The antibodies used for IP were as follows: Rabbit anti-PYK2 (Proteintech, Cat#17592-1-AP, 1:1500), Mouse anti-PYK2 (Cell Signaling Technology, 3480 S, 1:1500), Rabbit anti-Snail1 (Proteintech, Cat#13099-1-AP, 1:2000), Mouse anti-Snail1 (Cell Signaling Technology, #3895S, 1:2000), Phospho-Tyrosine Rabbit mAb, (Cell Signaling Technology, #8803, 1:3000).

### Immunofluorescent analysis

The transfected cells in the logarithmic growth stage were seeded onto cell slides and incubated overnight. When the cell density reached approximately 50%, they were fixed with a 4% paraformaldehyde solution and then treated with 0.1% Triton X-100. Afterward, the cells were washed with Phosphate Buffered Saline, and a blocking solution of 5% Bovine Serum Albumin (BSA) was added. The mixture was then left at room temperature for one hour. Afterwards, it was incubated with the corresponding antibody at 4 °C overnight. Fluorescent secondary antibodies were incubated in the dark at room temperature for one hour. After being washed with Phosphate Buffered Saline (PBS), the slides were covered and sealed with an antifade mounting medium containing DAPI. Subsequently, they were photographed under a laser confocal microscope (Ren et al. 2022). The antibodies used for Immunofluorescent analysis were as follows: Rabbit anti-PYK2 (Proteintech, Cat#17592-1-AP, 1:100), Mouse anti-PYK2 (Cell Signaling Technology, 3480 S, 1:100), Rabbit anti-Snail1 (Proteintech, Cat#13099-1-AP, 1:100), Mouse anti-Snail1 (Cell Signaling Technology, #3895S, 1:100).

### RNA extraction and real-time PCR

Total RNA was extracted using the Trizol Method, and cDNA was obtained by reverse transcribing with the cDNA synthesis kit (Takara Bio). Quantitative real-time PCR was then performed using SYBR Green PCR Master Mix (Takara) and the CFX96 Real-Time PCR detection system (Bio-Rad) (Lu et al. 2020). The primer sequences are provided in the supplementary material (Table S4).

### Animal experiments and establishment of mouse endometriosis model

All animal experiments have been approved by the Ethics Committee of Shandong Second Medical University and comply with the guidelines of the National Research Council. Thirty virgin female C57BL/6J mice, aged 5 weeks and weighed approximately (16–18 g), were purchased from the Laboratory Animal Center of Shandong Second Medical University (2022SDL254). All mice were acclimatized for one week before the start of the experiment, and the environment was maintained at a temperature of 20–26℃. The optimal relative humidity in the rearing room was 50-60%, and the humidity in the mouse rearing box was generally 5-10% higher than the environment. The mice had free access to standard rat chow and sterile water.

We model endometriosis in mice using allogeneic grafts. Donor mice (*n* = 10) were injected with estradiol benzoate via intramuscular injection, 3 µg/mouse, twice a week. After one week, all the donor mice were euthanized, dissected, and their uterine horn tissues were harvested. The uterine tissue is washed with normal saline and then cut into pieces of 1 mm^3^ in size using surgical scissors. The tissue fragments are mixed with lukewarm normal saline and injected into the abdominal cavity of the recipient mouse. To reduce unnecessary bias, the fragments from one donor mouse were evenly divided into two parts. One portion of the mixture was injected intraperitoneally into a mouse in the experimental group, while another portion was given to a mouse in the control group (Yan et al. 2019). Then the experimental group (*n* = 10) received an intraperitoneal injection of VS-6063 (50 mg/kg), and the control group (*n* = 10) was injected with Vehicle twice a week for three weeks. After three weeks, all mice were euthanized and examined for endometriotic lesions in the abdominal cavity.

### Statistical analysis

The in vitro experiments were conducted three or more times to ensure robustness of the results. Statistical analysis was performed using GraphPad Prism 9.0 software, employing methods such as the Mann–Whitney test, Student’s t-test, and one-way ANOVA. Pearson correlation analysis was utilized to assess the relationship between the two variables. Normally distributed data were tested using parametric tests, nonparametric tests, and Benjamini-Hochberg correction for multiple comparisons. Statistically significant differences were considered when p-values were less than 0.05, while values equal to or greater than 0.05 indicated no statistical significance. The finding did not achieve statistical significance (* *p* < 0.05, ** *p* < 0.01, *** *p* < 0.001, **** *p* < 0.0001).

## Results

### PYK2 enhances the ability of cells to proliferate and migrate in endometriosis

**Fig. 1:**
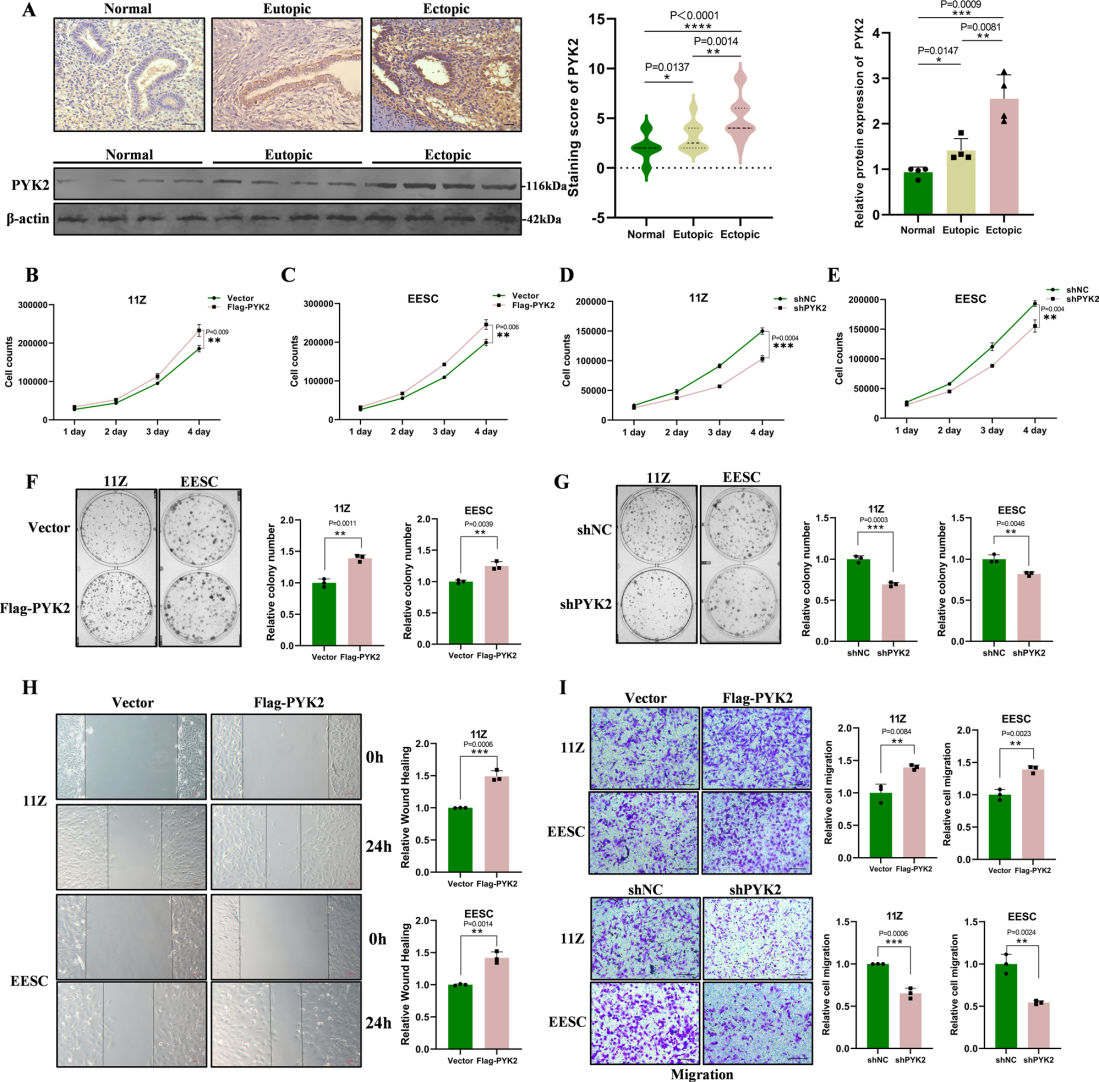
PYK2 enhances the ability of cells to proliferate and migrate in endometriosis. **A** The expression of PYK2 is different in different types of endometrial tissues. PYK2 protein levels are significantly elevated in endometriosis lesions compared to eutopic endometrium and normal endometrium. Representative IHC images show cytoplasmic staining intensity differences (scale bar, 20 μm). **B** and **C**. Overexpression of PYK2 promoted the proliferation of 11Z (**B**) and EESC (**C**) cells. **D** and **E**. Knockdown of PYK2 inhibited the proliferation of 11Z (**D**) and EESC (**E**) cells. **F** and **G**. The expression level of PYK2 had a significant effect on the clone-forming ability of both cells. **H**. Overexpression of PYK2 enhanced cell migration as demonstrated by scratch healing assays. **I**. By Transwell assay, the results revealed that the expression level of PYK2 had a significant effect on the migration ability of 11Z and EESC cells. (Scale bar, 20 μm). (All data represent mean ± SEM. The Mann–Whitney test, Wilcoxon test, and Spearman’s correlation analysis were used for data analysis. The Student’s t-test was used for data analysis. *P < 0.05, **P < 0.01, ***P < 0.001, ****P < 0.0001)

PYK2 has been shown to promote tumor cell proliferation and metastasis in a significant proportion of cancer types (Gil-Henn et al. 2023). Therefore, we examined the expression levels of PYK2 in clinical specimens obtained from patients with endometriosis. The results revealed significantly higher expression levels of PYK2 in the ectopic endometrial tissue samples compared to the normal and eutopic endometrial tissue samples (Fig. 1A). We constructed the corresponding plasmids and confirmed the successful expression or knockout in 11Z and EESC cells (Supplementary Fig. S1C). Cell proliferation assays demonstrated that enforcing PYK2 significantly enhanced cell proliferation (Fig. 1B, C), while knockdown of PYK2 inhibited the growth of 11Z and EESC cells (Fig. 1D, E). In addition, colony formation assays showed that the overexpression of PYK2 resulted in the formation of larger and more numerous colonies (Fig. 1F), whereas the knockdown of PYK2 impeded colony formation (Fig. 1G). We conducted scratch wound healing assays and transwell migration assays, which showed that the expression of PYK2 enhances the mobility of 11Z and EESC cells (Fig. 1H, I and Supplementary Fig. S1D). In summary, PYK2 promotes the proliferation, migration, and invasion of 11Z and EESC cells.

### PYK2 promotes EMT and inhibits decidualization in endometriosis

**Fig. 2:**
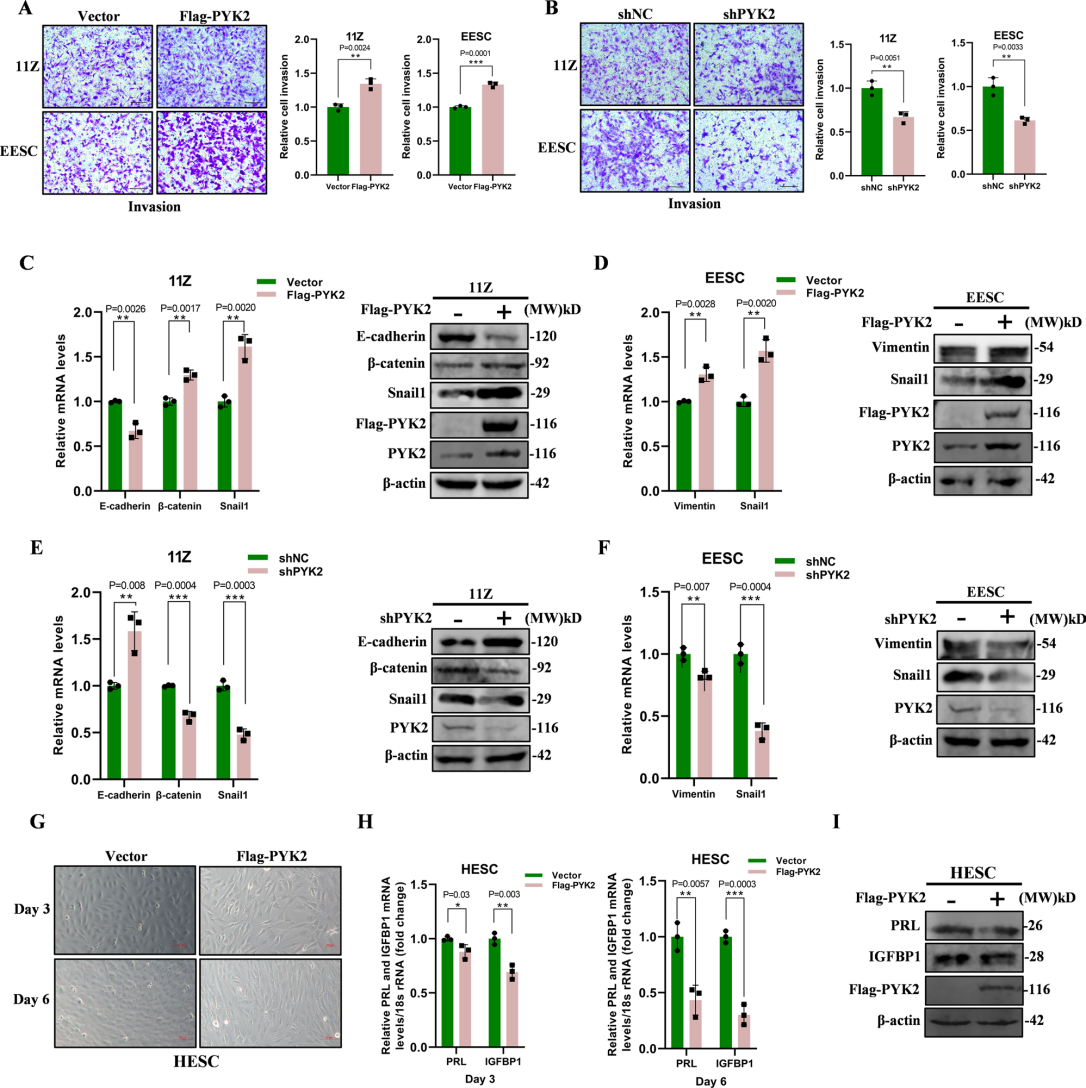
PYK2 promotes EMT and inhibits decidualization in endometriosis. **A**. Overexpression of PYK2 in 11Z and EESC cells was demonstrated by Transwell assay to result in increased invasion capacities, suggesting a role for PYK2 in enhancing cell invasion capacities (Scale bar, 20 μm). **B**. Knockdown of PYK2 in 11Z and EESC cells results in decreased invasion capacity, suggesting that PYK2 is necessary to maintain normal levels of invasion (Scale bar, 20 μm). **C** and **D**. Analysis of EMT-Related Factors After PYK2 Overexpression. Figures **C** and **D** display the mRNA and protein levels of EMT-related factors in 11Z (**C**) and EESC (**D**) cells after transfection with Flag-tagged PYK2. **E** and **F**. Detection of EMT-Related Factors After PYK2 Knockdown. In Figures **E** and **F**, the mRNA and protein levels of EMT-related factors in 11Z (**E**) and EESC (**F**) cells are shown after PYK2 knockdown. **G**. Cell microscopic images on days 3 and 6 of induced decidualization in vitro after PYK2 overexpression in HESC cells. **H**. The mRNA levels of PRL and IGFBP1 during decidualization after PYK2 overexpression. **I**. The protein levels of PRL and IGFBP1 during decidualization after PYK2 overexpression (All data represent mean ± SEM. The Student’s t-test was used for data analysis. *P < 0.05, **P < 0.01, ***P < 0.001, ****P < 0.0001)

EMT is not an all-or-nothing process; a crucial aspect of it is the acquisition of invasive and migratory abilities by epithelial cells (Konrad et al. 2020). PYK2 enhanced cell invasion capacity in vitro, as depicted in Figs. 2A-B. The results of immunohistochemical staining showed that the expression levels of EMT-related proteins were altered in endometriosis lesions (Supplementary Fig. S2A). E-cadherin, a glycoprotein responsible for maintaining cell-cell adhesion, is downregulated during the process of EMT (Canel et al. 2013). Snail1 also inhibits the expression of E-cadherin, leading to a decrease in intracellular domain (ICD) bound β-catenin. This release of β-catenin activates the Wnt/β-catenin signaling pathway and promotes downstream target gene expression (Kaufhold and Bonavida 2014). The mesenchymal cell marker Vimentin was upregulated during EMT, and immunohistochemical results showed that Vimentin was expressed in a small number of epithelial cells of ectopic endometrial tissue (Supplementary Fig. S2A), indicating that the epithelial cells had acquired mesenchymal characteristics (Yang et al. 2017; Cano et al. 2000; Furuya et al. 2017). Therefore, we investigated the changes in mRNA and protein levels of the aforementioned factors in 11Z and EESC cells upon overexpression of PYK2 (Fig. 2C, D). As shown in Figs. 2E-F, inhibition of PYK2 halted the progression of EMT.

Decidualization is a crucial process for the successful implantation and development of embryos. However, studies have shown that endometrial stromal cells from patients with endometriosis exhibit a decreased capacity for decidualization (Gellersen and Brosens 2014). Progestin induces decidualization and apoptosis of ectopic endometrial tissue. Co-treatment of cultures with progestin markedly enhances the cAMP response and allows sustained expression of the decidual phenotype in long-term cultures. Thus, cAMP signaling, via activation of the protein kinase A (PKA) pathway, sensitizes human endometrial stromal cells to progesterone (Klemmt et al. 2006). We have previously demonstrated that the expression of the PYK2 protein is slightly higher in eutopic endometrium compared to normal endometrium. Furthermore, we successfully induced decidualization of HESCs in vitro, which was evaluated based on morphological changes and levels of decidualization markers. Decidualization markers included IGFBP1 and PRL. IGFBP1 is actively involved in the regulation of decidualization. It can affect the morphological change and functional transformation of endometrial stromal cells and promote decidualization. PRL plays an important role in the maintenance of pregnancy. It can promote the growth and development of endometrium and provide a suitable environment for implantation and development of embryos. PRL also has the function of regulating immune function. During decidualization, PRL can affect the distribution and function of decidualized immune cells, thereby maintaining the immune tolerance state at the maternal-fetal interface. Following a 6-day induction period, control HESCs demonstrated complete decidualization transformation characterized by canonical morphological features of decidualization. In contrast, cells overexpressing PYK2 exhibited significantly attenuated decidualization (Fig. 2G). Similarly, overexpression of PYK2 inhibited the mRNA and protein levels of PRL and IGFBP1 (Fig. 2H, I), while upregulating its own mRNA and protein levels (Supplementary Fig. S2B). In conclusion, PYK2 promotes the development of endometriosis by enhancing epithelial-mesenchymal transition (EMT) and inhibiting decidualization. These mechanisms further contribute to infertility.

### PYK2 directly binds to Snail1

**Fig. 3:**
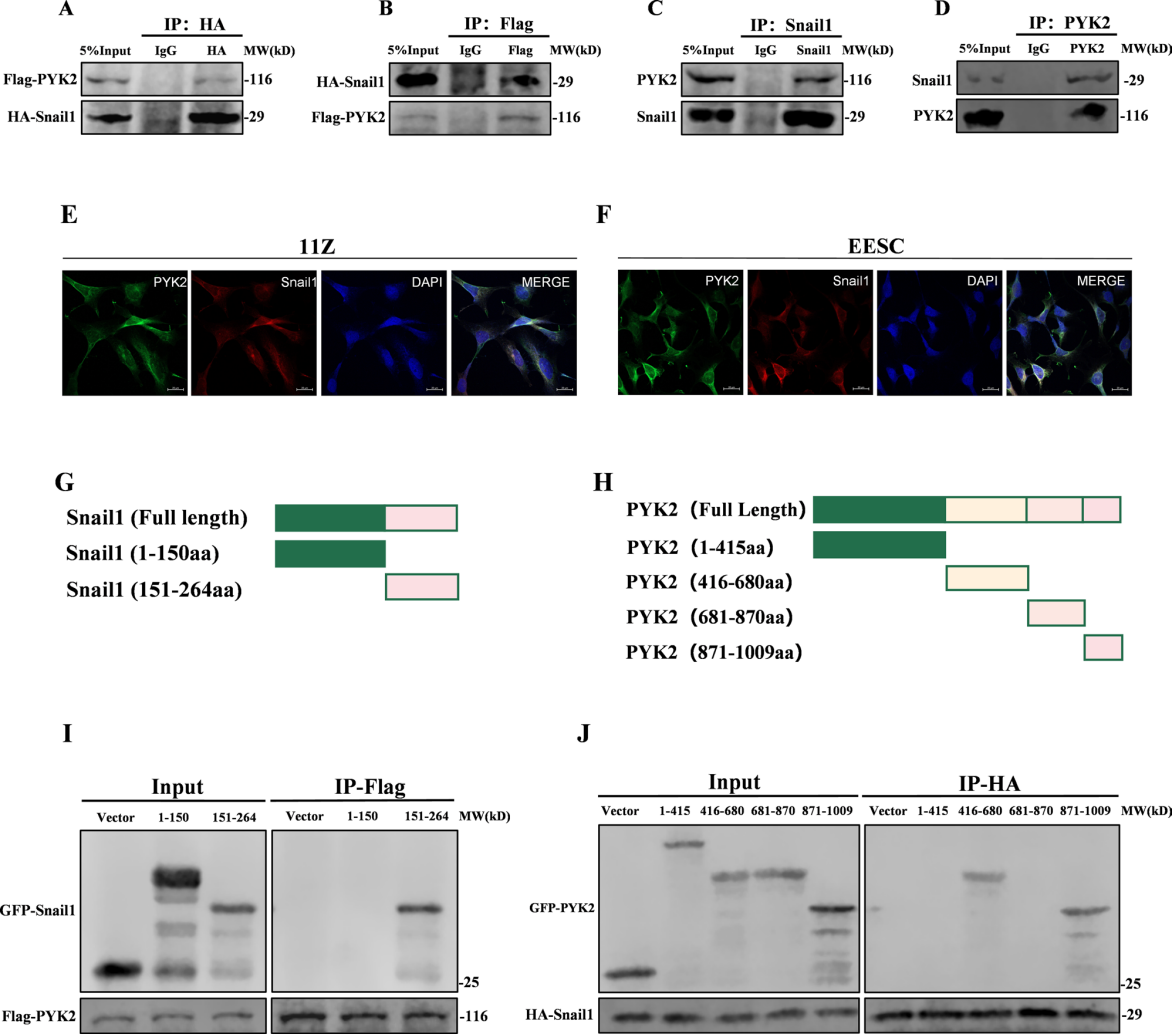
PYK2 directly binds to Snail1. **A** and **B**. Co-immunoprecipitation of HA-tagged Snail1 and Flag-tagged PYK2 in HEK293T cells. Anti-HA agarose was used for immunoprecipitation, followed by Western blot analysis (**A**). Anti-Flag agarose was used for immunoprecipitation, followed by Western blot analysis (**B**). The result shows an interaction between Snail1 and PYK2. **C**. Endogenous interaction between Snail1 and PYK2 in 11Z cells demonstrated by Snail1 immunoprecipitation (IP: Snail1). **D**. Reciprocal endogenous interaction confirmed by PYK2 immunoprecipitation (IP: PYK2) in 11Z cells. **E** and **F**. The localization of PYK2 and Snail1 in 11Z (**E**) and EESC (**F**) cells was analyzed by confocal immunofluorescence microscopy (Scale bar, 20 μm). **G** and **H**. Snail1 (**G**) and PYK2 (**H**) truncations diagrams. **I** and **J**. Interaction mapping between Snail1 truncations (HA-tagged) (**I**) and PYK2 domains (Flag-tagged) through reciprocal Co-IP. (The Mann–Whitney test, Wilcoxon test, and Student’s t-test were used for data analysis. All data represent mean ± SEM. *P < 0.05, **P < 0.01, ***P < 0.001, ****P < 0.0001)

Snail1, a C2H2 zinc-finger protein, is a well-established gene that regulates both the processes of epithelial-mesenchymal transition (EMT) and mesenchymal-epithelial transition (MET), playing a role in the pathogenesis of endometriosis (Paznekas et al. 1999). Our data demonstrate that Snail1 is highly expressed in ectopic endometriosis tissues (Supplementary Fig. S3A), which is consistent with a previous study (Wang et al. 2013). In our previous experiments, we observed a significant increase in the expression of Snail1 when PYK2 was overexpressed. Furthermore, there was a positive correlation between their expression levels in ectopic tissues (Supplementary Fig. S3B, C). Therefore, we wondered whether there was a correlation between the expressions of PYK2 and Snail1. To test this hypothesis, we co-transfected Flag-PYK2 and HA-Snail1 into HEK293T cells and observed their interaction through co-immunoprecipitation (Fig. 3A, B). Similarly, interaction between endogenous PYK2 and Snail1 was observed in 11Z cells (Fig. 3C, D). We analyzed the localization of PYK2 and Snail1 expression in both 11Z and EESC cells using confocal fluorescence microscopy. The results indicated that PYK2 and Snail1 co-localized in both the cytoplasm and nucleus (Fig. 3E, F). After analyzing the interaction between PYK2 and Snail1, we also identified their respective binding regions. We generated four truncated fragments of PYK2 and two truncated fragments of Snail1 (Fig. 3G, H). The binding sites were determined through Western blot analysis (Fig. 3I, J). Based on the aforementioned analysis, we concluded that PYK2 is a novel binding protein for Snail1.

### PYK2 increases the stability of Snail1 protein

**Fig. 4:**
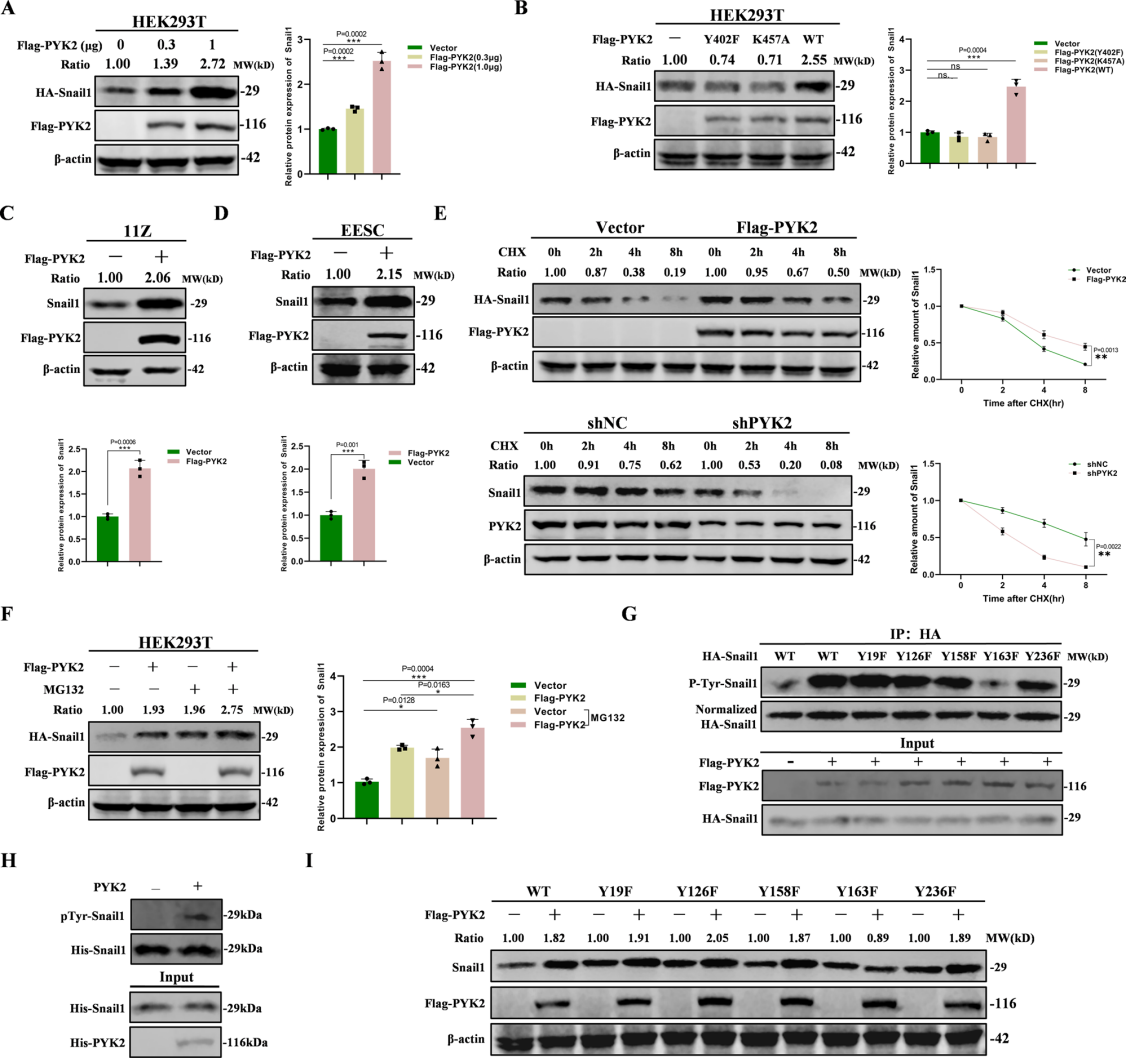
PYK2 increases the stability of Snail1 protein. **A**. After co-transfection of HA-Snail1 with gradient doses of Flag-PYK2 (0, 0.3, and 1 µg), Snail1 protein expression levels showed significant dose-dependent up-regulation. **B**. PYK2 wild-type (WT) co-transfection significantly enhanced Snail1 protein expression, which was lost in a kinase inactivation mutant (K457A) or an autophosphorylation site mutant (Y402F). **C** and **D**. Flag-tagged PYK2 was transfected with 11Z (**C**) and EESC (**D**) cells. The expression of Snail1 protein was analyzed by Western Blot. **E**. After co-transfection of HA-tagged Snail1 and Flag-tagged PYK2 in HEK293T cells and concurrent knockdown of PYK2 in 11Z cells, CHX (100 µmol/L) was applied to analyze the half-life of Snail1 protein. The results showed that the b expression of PYK2 affected the stability of Snail1 protein. **F**. HA-tagged Snail1 and Flag-tagged PYK2 were co-transfected into HEK293T cells, and the cells were treated with MG132 (100 µmol/L) for eight hours. The above cell extracts were subjected to SDS-PAGE analysis. PYK2 regulates Snail1 degradation through the ubiquitin-proteasome pathway. **G**. Results of transfection of Flag-tagged PYK2, wild-type Snail1, and different mutants after knockdown of Snail1 in 11Z cells and Co-immunoprecipitation using phosphorylated antibodies. Effect of Snail1 phosphorylation modification on its interaction with PYK2. **H**. Results of Western blot using the indicated antibodies after performing in vitro kinase assays. The results show the kinase activity of PYK2 against Snail1 or other substrates. **I**. After knockdown of Snail1 in 11Z cells, Flag-tagged PYK2, wild-type Snail1, and different mutants were transfected and Snail1 protein expression was analyzed by Western Blot. The results showed that these mutants had different effects on Snail1 protein expression. (All data represent mean ± SEM. The student’s t-test was used for data analysis. *P < 0.05, **P < 0.01, ***P < 0.001, ****P < 0.0001)

To delve deeper, we explored whether PYK2 influenced the abundance of Snail1. The results showed a positive correlation between the dose of PYK2 and the protein expression of Snail1 (Fig. 4A). Through its kinase activity, PYK2 promoted the protein stability of Snail1 (Fig. 4B). We subsequently demonstrated that the overexpression of PYK2 led to an increase in the protein levels of Snail1 in both 11Z and EESC cells (Fig. 4C, D). Similarly, knocking down PYK2 resulted in a decrease in the expression of Snail1 in both cell lines (Supplementary Fig. S4A, B). To investigate the role of PYK2 in regulating the stability of Snail1, we assessed the levels of Snail1 in transfected HEK293T cells treated with cycloheximide (CHX). The overexpression of PYK2 significantly enhanced the stability of Snail1, while the opposite effect was observed (Fig. 4E). We then investigated whether PYK2 regulates the stability of the Snail1 protein through the proteasome pathway in the presence of MG132. The data indicated that enforced PYK2 expression increased the stability of Snail1 (Fig. 4F). Next, we investigated the effect of PYK2 on Snail1 ubiquitination. Enforced expression of PYK2 suppressed Snail1 ubiquitination in the 11Z cells, while silencing of PYK2 promoted Snail1 ubiquitination (Supplementary Fig. S4C, D). We selected several upstream proteins of Snail1, including FBXL5, FBXO11 and FBXL14 of the F-box family, as well as CHIP/STUB1, a chaperone binding protein with E3 ubiquitin ligase activity (Park et al. 2019; Wu et al. 2015; Huang et al. 2023; Viñas-Castells et al. 2010). We verified that PYK2 may alter the expression level of Snail1 through CHIP, and that several other proteins did not change (Supplementary Fig. S4E-H). In general, PYK2 regulates the function of downstream proteins by phosphorylating them (Gil-Henn et al. 2023; Lee and Hong 2022). We then investigated whether PYK2 could phosphorylate Snail1. The specific phosphorylation site was identified by performing point mutations at the potential site of action of Snail1. PYK2 phosphorylated Snail1 at Y163 (Fig. 4G and Supplementary Fig. S4J). The results from the in vitro kinase assay were consistent (Fig. 4H). The data showed that PYK2 did not up-regulate the protein stability of Snail1 at Y163 (Fig. 4I). So, the above data demonstrates that PYK2 stabilizes the Snail1 protein by promoting its phosphorylation.

### Snail1 Y163 phosphorylation promotes cell proliferation, migration and invasion in 11Z and EESC cells

**Fig. 5:**
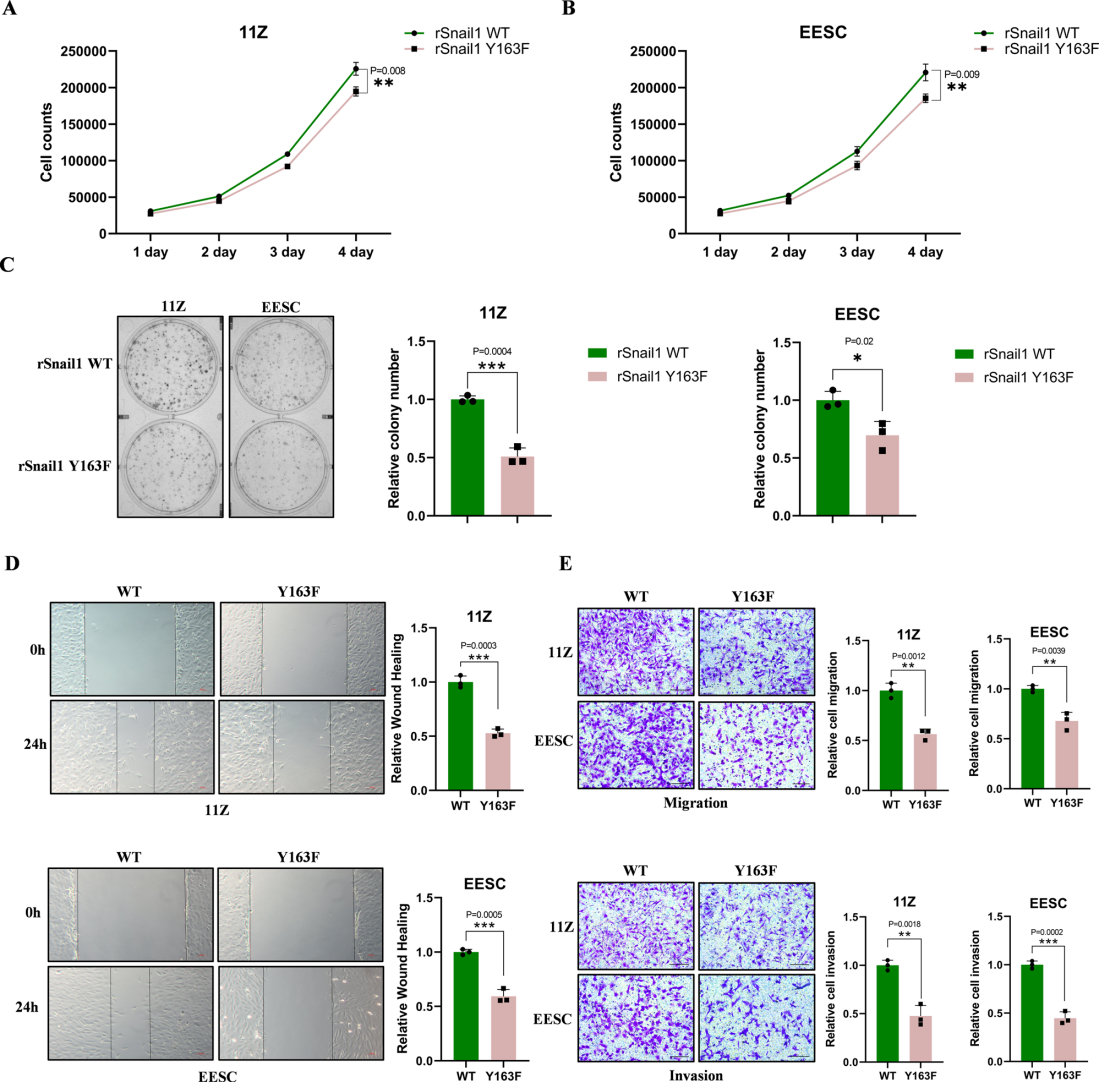
Snail1 Y163 phosphorylation promotes cell proliferation, migration and invasion in 11Z and EESC cells. **A** and **B**. Cell proliferation of rSnail1 (WT or Y163F) in 11Z (**A**) and EESC (**B**) cells. **C**. Clone formation of rSnail1 (WT or Y163F) in 11Z and EESC cells. **D**. After the forced expression of rSnail1 (WT or Y163F), the migration capacity of the cells was detected by scratch wound healing assay. **E**. The migration and invasion ability with Transwell assays of rSnail1 (WT or Y163F) in 11Z and EESC cells (Scale bar, 20 μm). (All data represent mean ± SEM. The student’s t-test was used for data analysis. *P < 0.05, **P < 0.01, ***P < 0.001, ****P < 0.0001)

To verify the effect of the Snail1 Y163 site on 11Z and EESC cells, we first knocked down the initial expression of Snail1 in 11Z and EESC cells, and then re-expressed it as shown above (Supplementary Fig. S4I). The Snail1 mutation Y163, as shown in Figs. 5A-B, inhibited the proliferation of 11Z and EESC cells. The clone formation assay results were consistent with this finding (Fig. 5C). Compared to wild-type Snail1, the Y163 mutation also inhibited the invasion and migration capacity of 11Z and EESC cells (Fig. 5D, E). Interestingly, the Snail1 Y163 mutation also inhibited the downstream expression of EMT-related proteins (Supplementary Fig. S4K). In summary, the phosphorylation of Snail1 at Y163 promotes biological behaviors in 11Z and EESC cells, such as cell proliferation and metastasis.

### PYK2 promotes Snail1 transcriptional activity

**Fig. 6:**
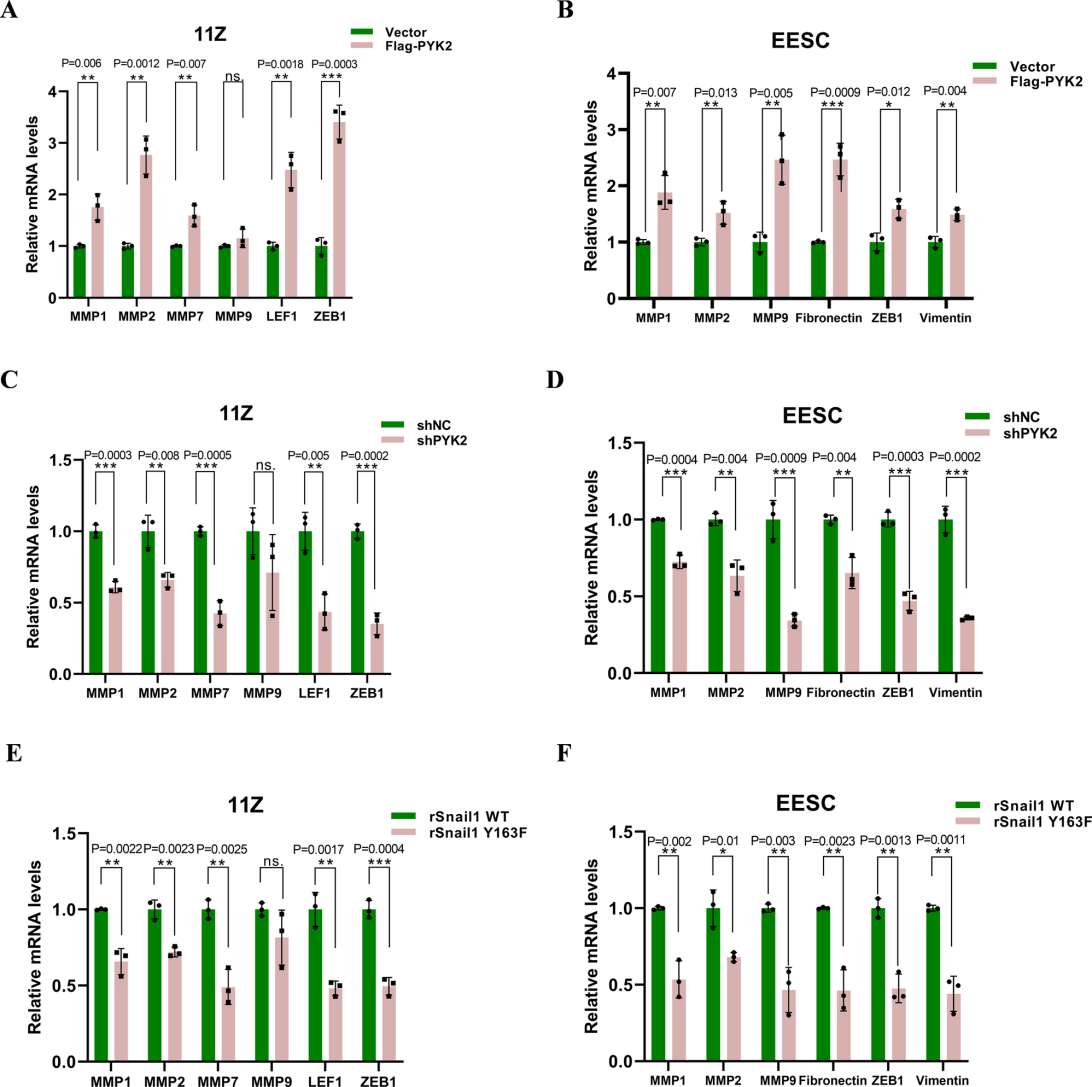
PYK2 promotes Snail1 transcriptional activity. **A** and **B**. The mRNA levels of Snail target genes were quantified by RT-PCR after transfection of PYK2 with Flag tag in 11Z (**A**) and EESC (**B**) cells. The effect of PYK2 overexpression on the expression of Snail target genes was demonstrated. C and D. After PYK2 was knocked down in 11Z (**C**) and EESC (**D**) cells, mRNA levels of Snail target genes were quantified by RT-PCR. The results demonstrated the effect of PYK2 knockdown on the expression of Snail target genes in 11Z and EESC cells. E and F. After knockdown of Snail1 in 11Z (**E**) and EESC (**F**) cells, the mRNA levels of Snail target genes were quantified by RT-PCR after re-expression of wild-type (WT) or Y163F mutated Snail1. The results demonstrated the effects of re-expressing different forms of Snail1 on the expression of Snail target genes in 11Z and EESC cells. (All data represent mean ± SEM. The student’s t-test was used for data analysis. *P < 0.05, **P < 0.01, ***P < 0.001, ****P < 0.0001)

The presence of Snail1 has been shown to increase the levels of Zinc finger E-box-binding homeobox 1 (ZEB1) protein during EMT (Guaita et al. 2002), and the expression of ZEB1 may serve as a potential indicator of invasive endometriosis (Furuya et al. 2017). Snail1 plays an irreplaceable role in regulating EMT, which leads to the upregulation of vimentin, fibronectin, and matrix metalloproteinases (MMPs) (Miyoshi et al. 2004). MMPs can degrade the extracellular matrix, alter cell-matrix adhesions, and promote tumor metastasis (Miyoshi et al. 2004). Lymphoid Enhancing Factor 1 (LEF1) is a 44.2 kDa protein that belongs to the TCF/LEF family, and it has been shown to play an essential role in the proliferation of endometrial glands (Shelton et al. 2012). Based on the theory mentioned above, we hypothesized that PYK2 could regulate the transcriptional activity of Snail1 and subsequently conducted relevant experiments. Results revealed that overexpression of PYK2 increased the mRNA levels of Snail1 target genes in 11Z cells (Fig. 6A) and EESC cells (Fig. 6B). The opposite effect was observed when PYK2 was knocked down (Fig. 6C, D). Interestingly, mutated Snail1 also caused changes in the mRNA levels of downstream target genes (Fig. 6E, F). Thus, PYK2 enhances the transcriptional activity of Snail1.

### PYK2 inhibitor VS-6063 has therapeutic effects in vitro and in vivo for endometriosis

**Fig. 7:**
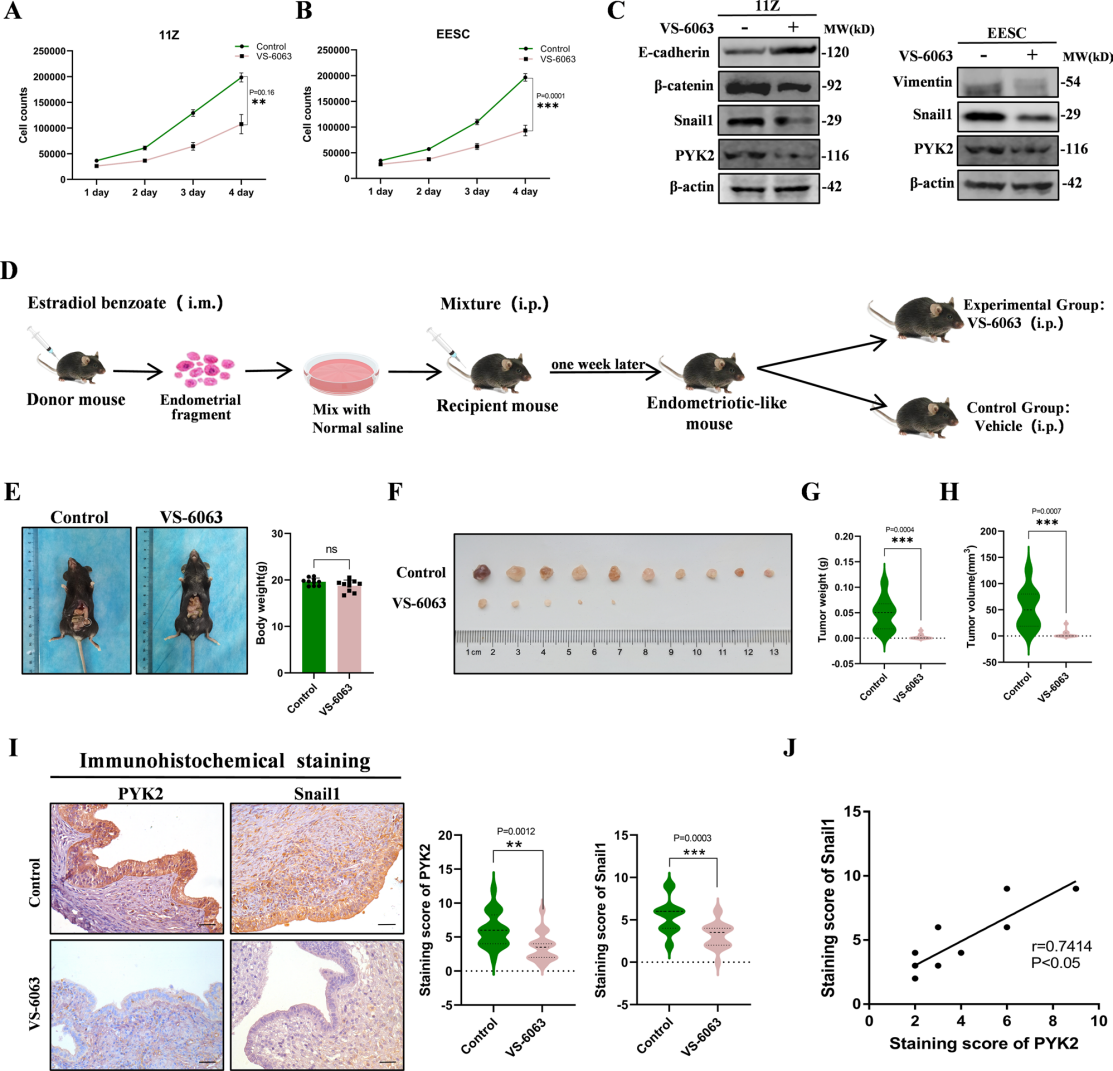
PYK2 inhibitor VS-6063 has therapeutic effects in vitro and in vivo for endometriosis. **A** and **B**. We examined the effect of VS-6063 on the proliferation ability of 11Z (**A**) and EESC (**B**) cells by cell counting. The results showed that the cell proliferation ability of the experimental group was significantly changed compared to the control group. **C**. The effect of VS-6063 on the protein expression levels of EMT-related factors in 11Z and EESC cells was analyzed by Western Blot. **D**. Endometriosis model was established using 5-week-old C57BL/6J female mice. **E**. Statistical analysis showed that there was no significant difference between the body weights of mice in the experimental and control groups. **F**, **G**, and **H**. The size of the ectopic tissue was observed and the volume and weight of the ectopic tissue were measured. The results showed that there were differences between the experimental and control groups in terms of ectopic tissue size, volume and weight. **I**. Immunohistochemical expression and comprehensive scores of PYK2 and Snail1 in two groups of endometriosis mouse models were demonstrated. The results showed that there were significant differences in the expression of PYK2 and Snail1 between the experimental and control groups (Scale bar, 20 μm). **J**. Pearson correlation analysis was performed on PYK2 and Snail1 staining scores. (All data represent mean ± SEM. The Student’s t-test was used for data analysis. *P < 0.05, **P < 0.01, ***P < 0.001, ****P < 0.0001)

VS-6063, also known as Defactinib or PF-04554878, is a novel FAK inhibitor developed by Pfizer. It is a highly potent dual inhibitor of FAK and PYK2 and has been successfully used in clinical trials for cancer treatment. VS-6063 exhibits high selectivity for both PYK2 and FAK (Lin et al. 2018; Gerber et al. 2020). It has been confirmed that FAK expression is increased in endometriotic lesions (Nagai et al. 2020). Since no suitable Pyk2-specific inhibitor is available, the effect of VS-6063 on the development of endometriosis was investigated both in vitro and in vivo. The IC_50_ values of VS-6063 were determined in two cell lines (Supplementary Fig. S5A). Subsequently, VS-6063 was applied to 11Z and EESC cells, resulting in the inhibition of cell proliferation, invasion, and migration (Fig. 7A, B and Supplementary Fig. S5B, C, D). The experimental results showed that VS-6063 could significantly promote decidualization process (Supplementary Fig. S5E, F, G). VS-6063 also reduced the expression of EMT-related proteins (Fig. 7C). To investigate the in vivo effect of VS-6063, we established a mouse model of endometriosis to examine its impact on the growth of endometriosis lesions (Fig. 7D). The results demonstrated that VS-6063 inhibited the development of endometriosis (Fig. 7E-H and Supplementary Fig. S6A). We then performed IHC staining to detect the expression of PYK2 and Snail1 in both groups. As expected, the expression levels of PYK2 and Snail1 were reduced after treatment with VS-6063 (Fig. 7I). Furthermore, a positive correlation was observed between the expressions of PYK2 and Snail1 in mouse endometriosis tissues (Fig. 7J). Additionally, we found alterations in the expression levels of proteins associated with EMT and proliferation as well (Supplementary Fig. S6B). In summary, the PYK2 inhibitor was able to inhibit the proliferation, migration, and invasion of 11Z and EESC cells in vitro. Furthermore, it exhibited therapeutic effects on endometriosis lesions in mice. It is reasonable to speculate that VS-6063 inhibits the development of endometriosis.

## Discussion

Endometriosis is an ongoing disease that can result in progressively increasing pain and infertility. Infertility caused by endometriosis may be attributed to pelvic adhesion, organ infiltration, and tissue fibrosis, leading to changes in anatomical structure (58). In a large cohort study of women of reproductive age, it was found that women under 35 years old with endometriosis had twice the risk of infertility compared to those without endometriosis (Prescott et al. 2016). This increased risk may be associated with reduced ovarian reserve and decreased endometrial receptivity due to endometriosis. Therefore, it is necessary to investigate the pathological mechanisms underlying endometriosis. EMT is involved in the pathogenesis of endometriosis, and Snail1, which plays a central role in EMT, has also been shown to be highly expressed in endometriosis tissue (Wang et al. 2013). However, the mechanism by which PYK2 regulates Snail1 remains unclear.

In our study, we demonstrate that PYK2 is a contributing factor in the development of endometriosis. We find a positive correlation between the expression levels of PYK2 and the proliferative, migratory, and invasive abilities of 11Z and EESC cells. PYK2 interacts with Snail1 and promotes protein stability by phosphorylating Snail1 at the Y163 site, thereby promoting epithelial-mesenchymal transition (EMT) in endometriosis. This is supported by the following experimental results. First, we find that high levels of PYK2 promote the proliferation, migration and invasion of 11Z and EESC cells. Overexpression of PYK2 increases breast cancer cell migration (Al-Juboori et al. 2019). Second, Co-IP assays confirm the protein-protein interaction between the two, and we performed a point mutation in the potential action site of Snail1, which reveals that PYK2 phosphorylates Snail1 at Y163. Previous studies have shown that Snail1 triggers EMT by up-regulating MMP2 and MMP9. Furthermore, both Snail1 and Slug can maintain EMT through continuous stimulation of MMP9 (Qiao et al. 2010). Therefore, we searched for Snail1 target genes and found that mRNA levels of these genes increase when PYK2 is overexpressed. However, after the point mutation compared to the wild type, there was a decrease in mRNA levels of Snail1 target gene. Finally, we utilized a PYK2 inhibitor called VS-6063 and demonstrated that inhibiting the expression of PYK2 can effectively suppress the development of endometriosis both in vivo and in vitro.

Decidualization of the endometrium is necessary for embryo implantation, growth and development (Owusu-Akyaw et al. 2019). Inadequate decidualization response of the endometrium is also a cause of infertility in patients with endometriosis (Pathare et al. 2023). Consistent with our study findings, excessive expression levels of PYK2 in endometriosis suppressed the expression of decidualization-related markers. However, due to the limitations in our study methodology, we did not conduct an in-depth investigation into how PYK2 inhibits decidualization, which is also a limitation of this study. We hope to elucidate this mechanism in subsequent studies.

Taken together, our study has identified Snail1 as a novel substrate for PYK2. By phosphorylating the Snail1 protein, PYK2 promotes cell proliferation and epithelial-mesenchymal transition (EMT) in endometriosis, thereby enhancing the stability of Snail1 protein. Additionally, treatment with VS-6063 inhibits the growth of endometriosis lesions in vivo. These findings suggest that targeting PYK2 may hold potential for the treatment of endometriosis.

## Conclusions

We identified PYK2 as a novel binding partner of Snail1, and found that PYK2 up-regulates the expression of Snail1. This suggests that PYK2 promotes the occurrence and development of endometriosis by increasing Snail1 levels, making it a potential therapeutic target for endometriosis.

### Strengths and weaknesses

Our study identifies PYK2 as a novel substrate and binding partner of Snail 1 in endometriosis. This finding expands our understanding of the molecular mechanisms of this disease. By demonstrating that PYK2 phosphorylates Snail1, we mechanistically revealed how PYK2 promotes cell proliferation and epithelial-mesenchymal transition (EMT) in endometriosis. Targeting PYK2 may have therapeutic potential for the treatment of endometriosis, as inhibition of PYK2 with VS-6063 suppressed the growth of endometriosis lesions in vivo. Although we discovered the role of PYK2 in endometriosis, we did not study it in depth. This is a limitation of our study and an area that needs to be further explored in subsequent studies. Although PYK2 was identified as a potential therapeutic target, more studies are needed to fully elucidate the mechanisms by which PYK2 promotes endometriosis and to validate the efficacy and safety of targeting PYK2 in a clinical setting.

## Electronic supplementary material

Below is the link to the electronic supplementary material.

Supplementary Material 1

Supplementary Material 2

## Data Availability

No datasets were generated or analysed during the current study.

## Abbreviations

EMT: Epithelial-mesenchymal transition
PYK2/PTK2B: Proline-rich tyrosine kinase 2
ESHRE: European Society of Human Reproduction and Embryology
CPSP: Chronic post-surgical pain
AWE: Abdominal wall endometriosis
ECM: Extracellular matrix
FAK: Focal adhesion kinase
CCL18: Chemokine ligand 18
HESC: Human endometrial stromal cell
EESC: Ectopic endometrial stromal cell
11Z: Endometrial epithelium cell
BSA: Bull Serum Albumin
PBS: Phosphate Buffered Saline
α-SMA: α-smooth muscle actin
PRL: Prolactin
IGFBP1: Insulin-like growth factor binding protein 1
E-cadherin: Epithelial cadherin
MET: Mesenchymal-epithelial transition
CHX: Cycloheximide
ZEB1: Zinc finger E-box-binding homeobox 1
MMPs: Matrix metalloproteinases
LEF1: Lymphoid Enhancing Factor 1
PTX: Paclitaxel
