# Supplementary material for: PYK2 promotes cell proliferation and epithelial-mesenchymal transition in endometriosis by phosphorylating Snail1
Source: Mol Med. 2025 Apr 27;31:155. doi: 10.1186/s10020-025-01218-1 (PMC12036249; doi:10.1186/s10020-025-01218-1)
Supplement: Supplementary file 1 — Supplementary Material 1 [file 10020_2025_1218_MOESM1_ESM.docx]

**Table S1. Inclusion criteria for clinical specimens**

| **The endometriosis group** | **Control group** |
| --- | --- |
| Women of childbearing age (22-40 years) | Women of childbearing age (22-40 years) |
| Normal menstrual cycles (25-31 days) | Normal menstrual cycles (25-31 days) |
| Patients with endometriosis diagnosed by ultrasound or laparoscopy underwent surgical intervention, with the diagnosis being histopathologically confirmed through postoperative pathology. Lesional tissue was collected by curettage, hysteroscopic biopsy, diagnostic curettage, negative pressure suction curettage, and endometrial aspiration biopsy. | Patients without endometriosis, endometrial adenomyosis, or other endometrial abnormalities diagnosed by laparoscopy, who sought assisted reproductive technology due to male infertility. Endometrium was collected by curettage, hysteroscopic biopsy, diagnostic curettage, negative pressure suction curettage, and endometrial aspiration biopsy. |
| No use of hormonal drugs in the past 3 months | No use of hormonal drugs in the past 3 months |
| Endocrine diseases, autoimmune diseases and coagulopathy-related diseases were excluded | Endocrine diseases, autoimmune diseases and coagulopathy-related diseases were excluded |
| Patients with gynecological disorders such as fibroids, polycystic ovary syndrome, adenomyosis, and uterine polyps are excluded by ultrasonography, magnetic resonance imaging (MRI), hysterosalpingography, and pathology. | Patients with gynecologic diseases such as fibroids, polycystic ovary syndrome, adenomyosis, and uterine polyps are excluded by ultrasonography, MRI, hysterosalpingography, and pathology |

**Table S2. The characteristics of the recruited subjects**

|  | **Control group** | **The endometriosis group** |
| --- | --- | --- |
| No of case | 20 | 20 |
| Age (mean ±SD) | 31.7±5.2 | 31.0 ±5.1 |
| Menstrual cycle phase |  |  |
| Proliferative stage | 15 | 17 |
| Secretory phase | 5 | 3 |
| rASRM stag |  |  |
| III | - | 8 |
| IV | - | 12 |
| Type |  |  |
| Peritoneal endometriosis | - | 6 |
| Ovarian endometriosis | - | 14 |

**Table S3. shRNA sequences used in this study.**

| **shRNA** | **Sense (5’-3’)** | **Anti-sense (5’-3’)** |
| --- | --- | --- |
| shRNA-Control | TTCTCCGAACGGTCACGT | ACGTGACCGTTCGGAGAA |
| shRNA1-PYK2 | CGTATCCTCAAGGTCTGCTTC | GAAGCAGACCTTGAGGATACG |
| shRNA1-Snail1 | GCAGGACTCTAATCCAGAGTT | AACTCTGGATTAGAGTCCTGC |

**Table S4. Primary antibodies, plasmids and reagents used in this study.**

| **REAGENT or RESOURCE** | **SOURCE** | **IDENTIFIER** |
| --- | --- | --- |
| **Antibodies** | | |
| Mouse anti-HA | Sigma-Aldrich | Cat#H3663 |
| Mouse anti-Flag | Sigma-Aldrich | Cat#F1804 |
| Mouse anti-β-actin | Sigma-Aldrich | Cat#A1978 |
| Rabbit anti-HA | Proteintech | Cat#51064-2-AP |
| Rabbit anti-Flag | Proteintech | Cat#20543-1-AP |
| Rabbit anti-β-actin | Proteintech | Cat#20536-1-AP |
| Rabbit anti-PYK2 | Proteintech | Cat#17592-1-AP |
| Mouse anti-PYK2 | Cell Signaling Technology | #3480S |
| Mouse anti-GFP | Sigma-Aldrich | Cat#G6539 |
| Rabbit anti-PRL | Proteintech | Cat#16525-1-AP |
| Rabbit anti-IGFBP1 | Proteintech | Cat#13981-1-AP |
| Rabbit anti-Cytokeratin 7 | Proteintech | Cat#15539-1-AP |
| Rabbit anti-GFP | Proteintech | Cat#50430-2-AP |
| Rabbit anti-Ubiquitin | Proteintech | Cat#10201-2-AP |
| Rabbit anti-Vimentin | Proteintech | Cat#10366-1-AP |
| Rabbit anti-E-cadherin | Proteintech | Cat#20874-1-AP |
| Rabbit anti-α-SMA | Proteintech | Cat#14395-1-AP |
| Rabbit anti-β-catenin | Proteintech | Cat#51067-2-AP |
| Rabbit anti-Snail1 | Proteintech | Cat#13099-1-AP |
| Mouse anti-Snail1 | Cell Signaling Technology | #3895S |
| Rabbit anti-Ki67 | Proteintech | Cat#27309-1-AP |
| Normal mouse IgG | Santa Cruz Biotechnology | Lot#L2721 |
| Phospho-Tyrosine Rabbit mAb | Cell Signaling Technology | #8803 |
| IRDye 800CW goat anti-rabbit | LI-COR | Cat#925-32210 |
| IRDye 680LT goat anti-mouse | LI-COR | Cat#925-68020 |
| Anti-HA Affinity Gel | Sigma-Aldrich | Cat#E6779 |
| Anti-FLAG Affinity Gel | Sigma-Aldrich | Cat#F2426 |
| Cell lysis buffer | Beyotime | P0013G |
| Cycloheximide | MedChemExpress | Cat#HY-12320 |
| MG132 | MedChemExpress | Cat#HY-13259 |
| Lipofectamine 2000 | Thermo Fisher | Cat#11668019 |
| 17β-Estradiol | MedChemExpress | HY-B0141 |
| progesterone | MedChemExpress | HY-N0437 |
| cAMP | MedChemExpress | HY-B1511 |
| paraformaldehyde | Beyotime | P0099 |
| IV collagenase | Sigma-Aldrich | C4-BLOC |
| DAB | MedChemExpress | HY-W025920 |
| **Bacterial Strain** | | |
| E. coli DH5α | Thermo Fisher | Cat#18258012 |
| E. coli Stable 3 | Thermo Fisher | Cat#C737303 |
| E. coli BL21 | Thermo Fisher | Cat#C600003 |
| **Recombinant DNA** | | |
| pCDNA3.0/neo-HA-PYK2 | This paper | N/A |
| pCDNA3.1/neo-Flag-PYK2 | This paper | N/A |
| pCDNA3.1/neo-Flag-PYK2 (mutants) | This paper | N/A |
| pCDNA3.0/neo-HA-Snail1 | This paper | N/A |
| pCDNA3.0/neo-HA-Snail1 (mutants) | This paper | N/A |
| pCDNA3.0/neo-Flag-Snail1 | This paper | N/A |
| pEGFP-C1-PYK2 (P1, P2, P3 or P4) | This paper | N/A |
| pEGFP-C1-Snail1(S1 or S2) | This paper | N/A |
| pLVX-shRNA1-PYK2 | This paper | N/A |
| pLVX-shRNA1-Snail1 | This paper | N/A |
| pLVX-IRES-neo-Snail1 (WT or mutant) | This paper | N/A |

**Table S5. The primers used for real-time PCR in this study.**

| **Gene** | **Forward** | **Reverse** |
| --- | --- | --- |
| E-cadherin | GACAACAAGCCCGAATT | TTAGAACCCGATCCAACAGC |
| β-catenin | GTGCTGAAGGTGCTATCTGTCTGC | TGAACAAGACGTTGACTTGGATCTG |
| Snail1 | TGCGTCTGCGGAACCTG | GGACTCTTGGTGCTTGTGGA |
| Vimentin | GAGAACTTTGCCGTTGAAGC | GCTTCCTGTAGGTGGCAATC |
| Fibronectin | CCACCCCCATAAGGCATA | GTAGGGGTCAAAGCACGAGTC |
| PRL | GAGGAGCAAACCAAACGGCTTC | AAGGCGAGACTCTTCATCAGCC |
| IGFBP1 | CCAAACTGCAACAAGAATG | GTAGACGCACCAGCAGAG |
| LEF1 | TGGCAGCCCTATTTCAGTTT | CAAAGGCTGTGCTTGCTTTT |
| MMP1 | GGGGCTTTGATGTACCCTAGC | TGTCACACGCTTTTGGGGTTT |
| MMP2 | CTGCATCCAGACTTCCTCAG | TCCTGGCAATCCCTTTGTATGT |
| MMP7 | ATGGGGAACTGCTGACATCAT | CCAGCGTTCATCCTCATCGAA |
| MMP9 | CGCAGACATCGTCATCCAGT | AACCGAGTTGGAACCACGAC |
| ZEB1 | AAGTGGCGGTAGATGGTA | TTGTA GCGACTGGATTTT |
| 18S | GTTGAACCCCATTCGTGATG | GCCTCACTAAACCATCCAA |
