# Supplementary material for: PYK2 promotes cell proliferation and epithelial-mesenchymal transition in endometriosis by phosphorylating Snail1
Source: Mol Med. 2025 Apr 27;31:155. doi: 10.1186/s10020-025-01218-1 (PMC12036249; doi:10.1186/s10020-025-01218-1)
Supplement: Supplementary file 2 — Supplementary Material 2 [file 10020_2025_1218_MOESM2_ESM.docx]

**
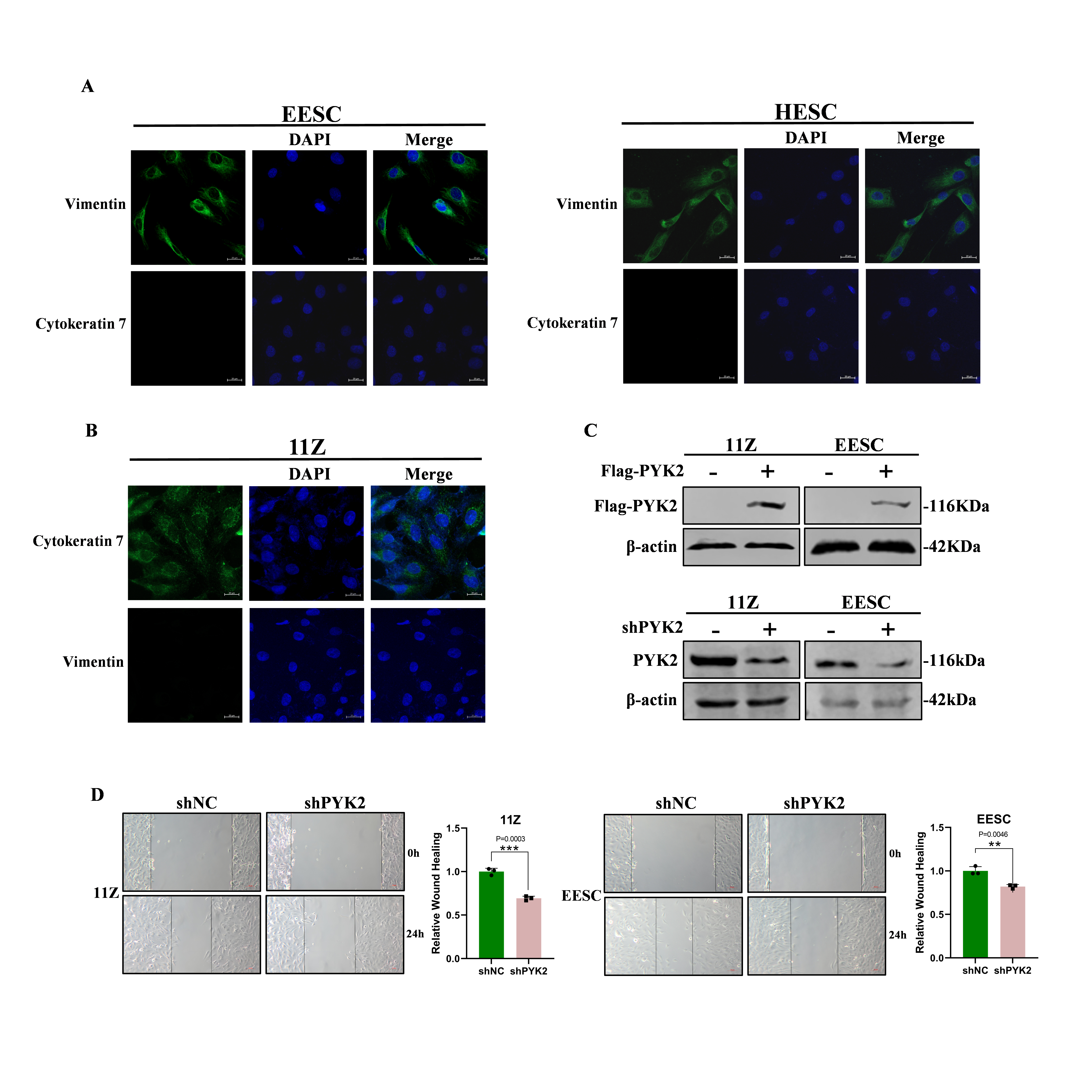
Figure S1. PYK2 promotes migration of 11Z and EESC cells, related to Figure 1**

(A) EESC and HESC cells were identified by immunofluorescent staining for Cytokeratin7 and Vimentin (Scale bar, 20µm). (B) 11Z cells were identified by immunofluorescent staining for Cytokeratin7 and Vimentin (Scale bar, 20µm). (C) Protein expression was significantly increased after overexpression of PYK2 in 11Z cells compared to the empty vector group. Protein expression was significantly reduced after knockdown of PYK2 in 11Z cells compared to controls. (D) Cell migration ability detected by scratch healing assay after reducing PYK2 expression.(All data represent mean ± SEM. The Student’s t-test was used for data analysis.*P<0.05, **P<0.01, ***P<0.001, ****P<0.0001)

**
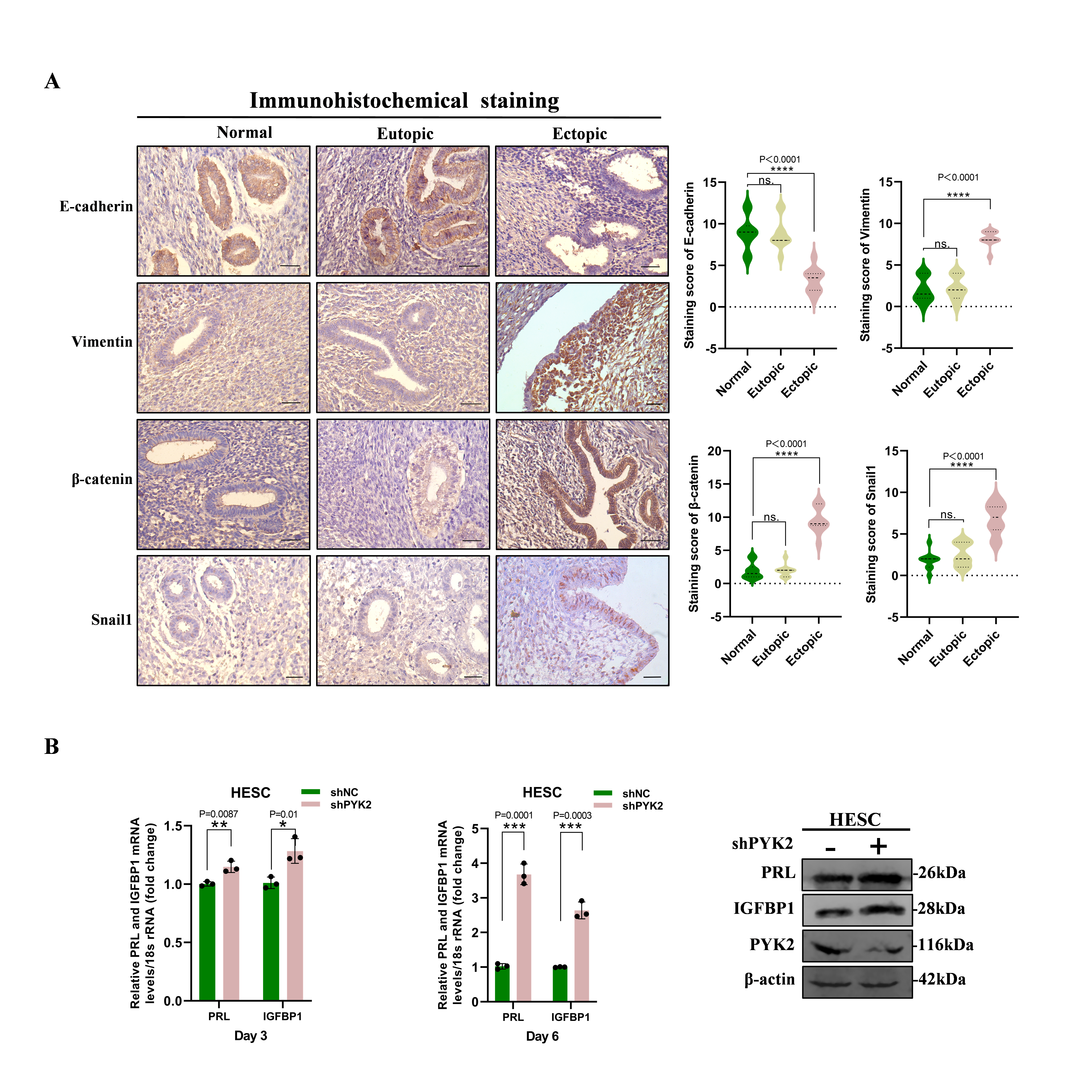
Figure S2. Expression levels of EMT-related proteins in endometriotic lesions and PYK2 inhibits decidualization, related to Figure 2**

(A) Figure A demonstrates representative staining photographs of E-cadherin, Vimentin, β-catenin, and Snail1 in normal endometrium, in situ endometrium, and ectopic endometrium samples. By comparing the intensity and distribution of staining in each group of samples, it was possible to observe the differences that existed between normal endometrium and eutopic endometrium and ectopic endometrium. (Scale bar, 20µm). (B) During decidualization in vitro, the mRNA levels and protein levels of PRL and IGFBP1 in HESC were changed after the expression of PYK2 was reduced on days 3 and 6. (All data represent mean ± SEM. The Student’s t-test was used for data analysis.*P<0.05, **P<0.01, ***P<0.001, ****P<0.0001)

**
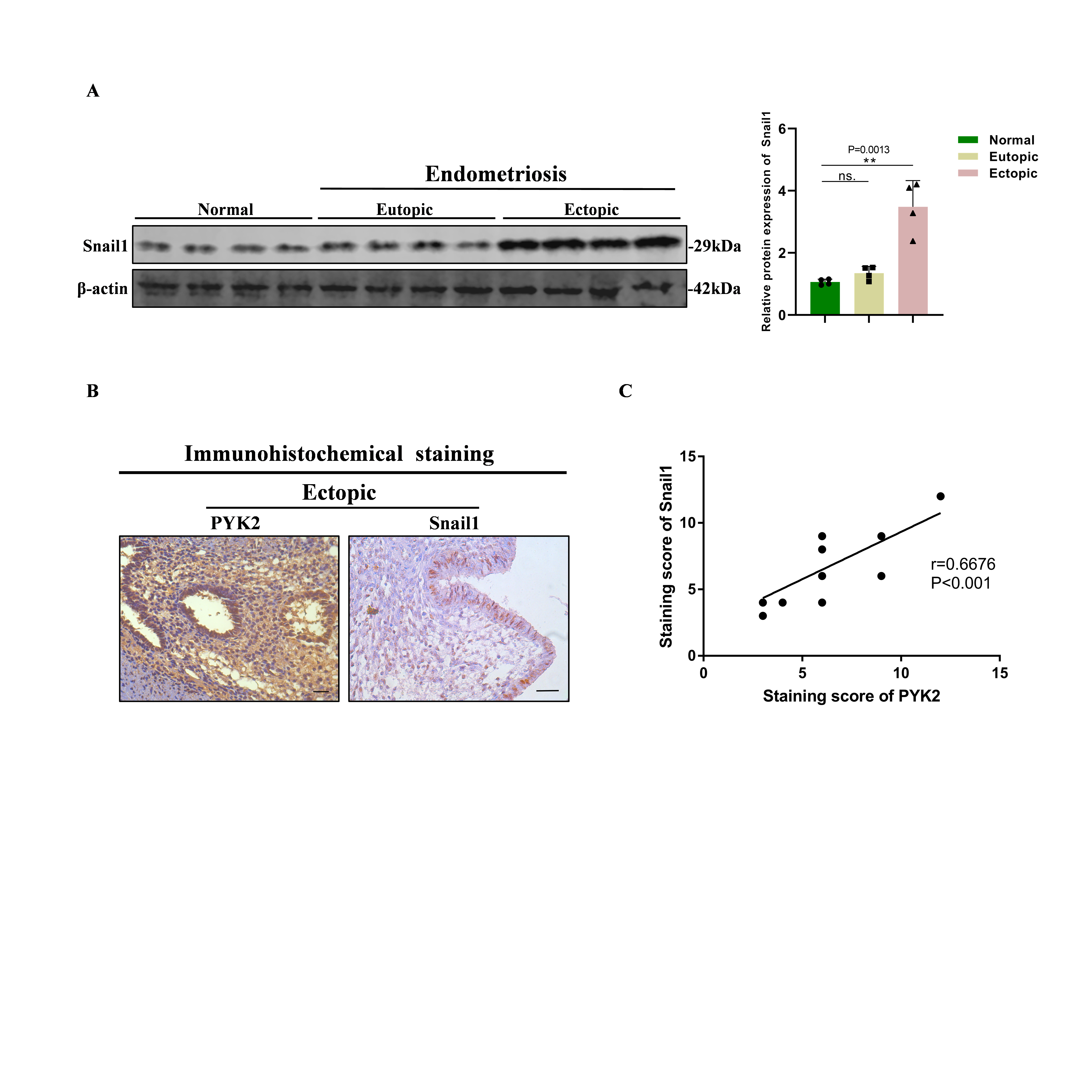
Figure S3. PYK2 expression is positively correlated with Snail1 in endometriosis lesions, related to Figure 3**

(A) The expression levels of Snail1 in normal endometrial tissue samples, eutopic endometrium from patients with endometriosis and endometriosis lesion samples were compared by Western Blot. (B) PYK2 and Snail1 proteins were highly expressed in endometriotic tissues. (C) Pearson correlation analysis was performed on PYK2 and Snail1 staining scores. (The Student’s t-test was used for data analysis. All data represent mean ± SEM. *P<0.05, **P<0.01, ***P<0.001, ****P<0.0001)

**
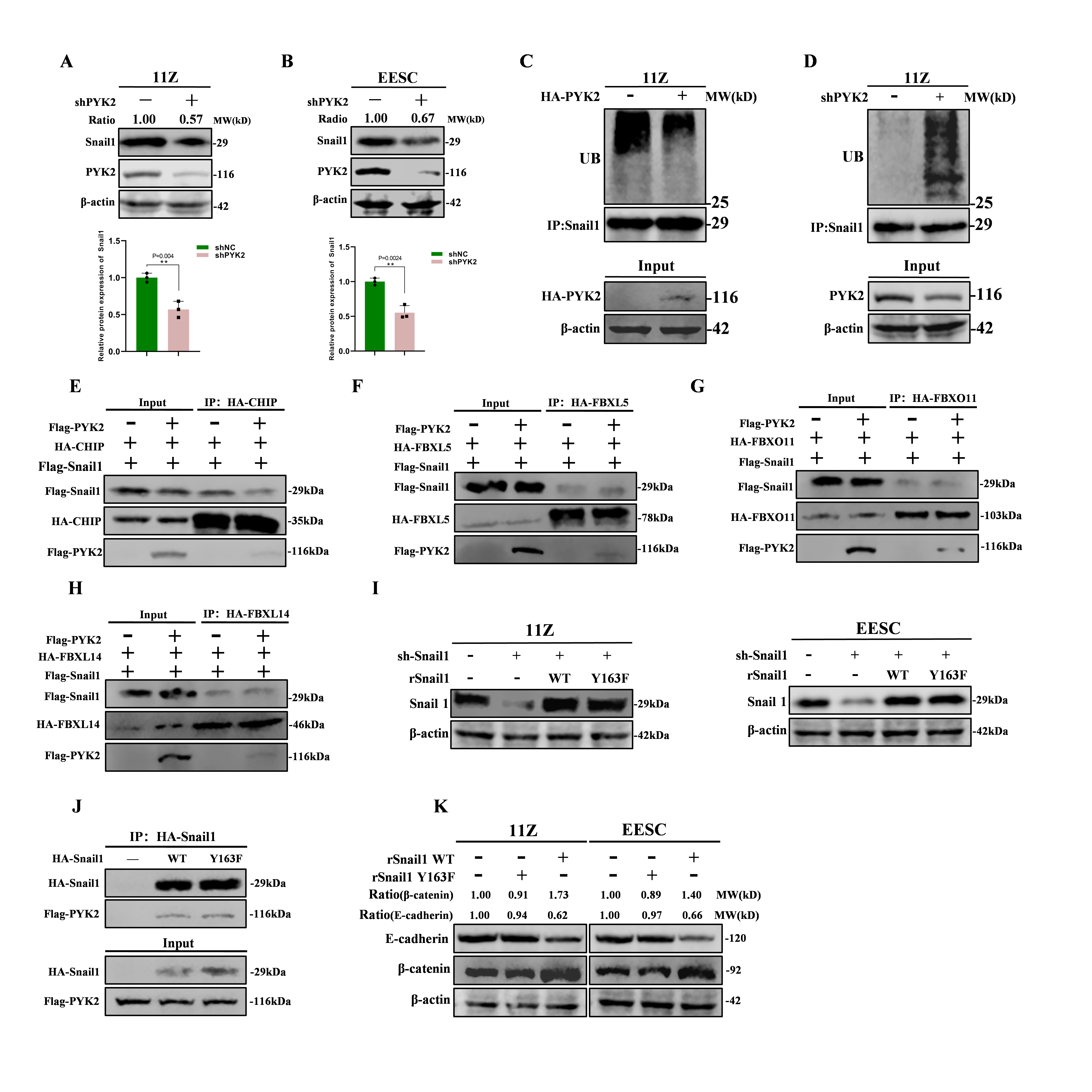
Figure S4. PYK2 promotes the protein stability of Snail1 and phosphorylates Snail1 at Y163, related to Figure 4 and Figure 5**

(A and B) PYK2 was knocked down in 11Z (A) and EESC (B) cells. The corresponding antibody was applied, Western Blot was used to analyze the expression of Snail1 protein. (C and D) Ubiquitination assays of endogenous Snail1 in the lysates from 11Z cells transfected with Flag-PYK2 (C) or stably expressing PYK2 shRNA (D). (E-H) Co-immunoprecipitation assay confirmed that PYK2 may change the expression level of Snail1 through CHIP (E), but FBXL5 (F), FBXO11 (G) and FBXL14 (H) did not change. (I) Snail1 was knocked out by shSnail1, and then re-expressed in 11Z and EESC cells. (J) Figure J demonstrates the results of co-transfection experiments in HEK293T cells. HA-Snail1 or HA-Snail1 Y163F were co-transfected with FLAG-PYK2, respectively. immunoprecipitation was performed with anti-Flag agarose followed by Western blot analysis (K) Results of protein expression levels of Snail1 downstream target genes analyzed by Western Blot after expression of wild-type Snail1 and Snail1 Y163F mutant in 11Z and EESC cells. (The Student’s t-test was used for data analysis. All data represent mean ± SEM. *P<0.05, **P<0.01, ***P<0.001, ****P<0.0001)

**
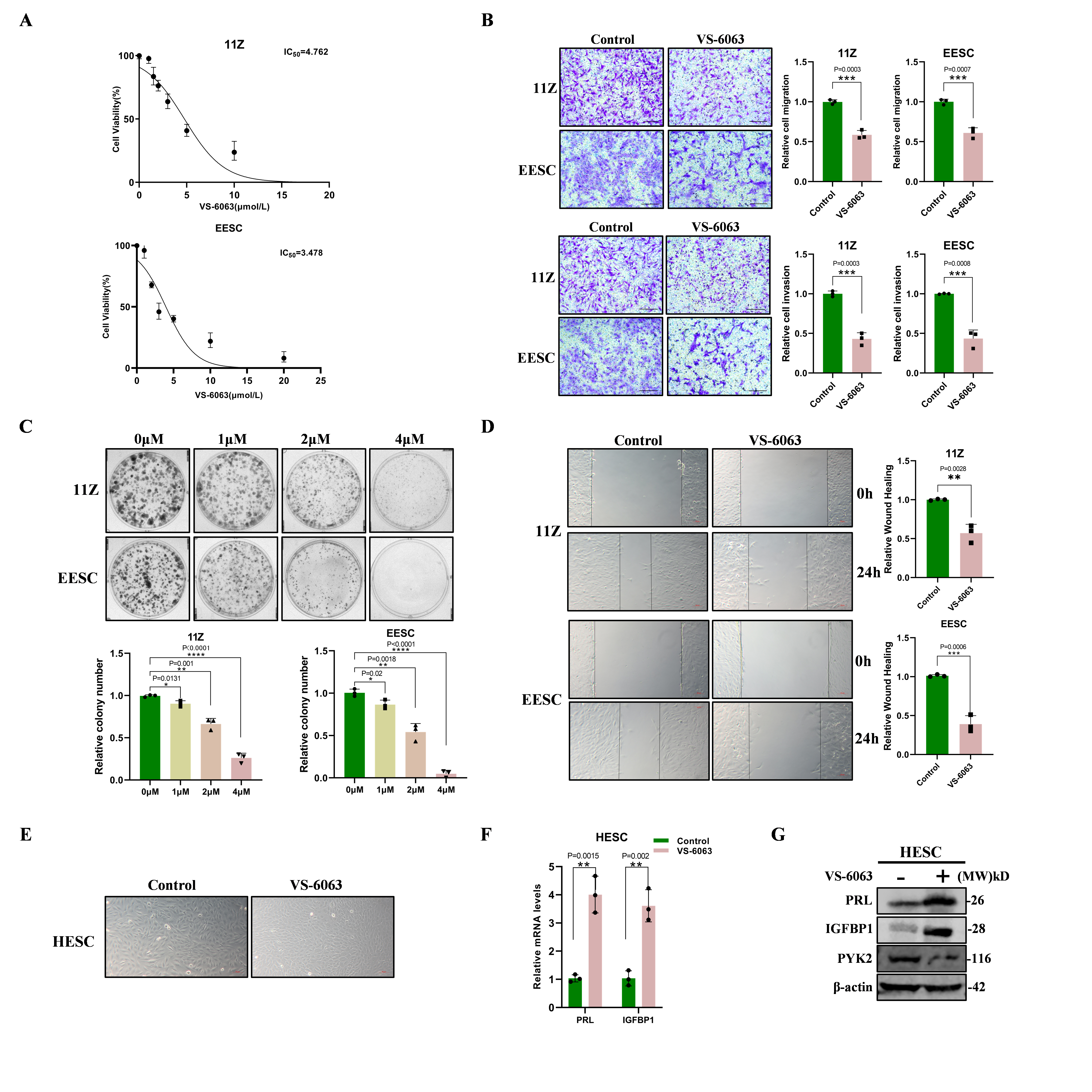
Figure S5. VS-6063 have therapeutic effects on endometriosis in vitro, related to Figure 7**

(A) VS-6063 acted on both cell lines in a concentration-dependent manner and the IC_50_ values of the drug in both cells were determined by cell counting methods. (B) The cells in the experimental group were treated with VS-6063 and the migratory capacity was compared with the control group at 24 hours after treatment. (C) Effect of VS-6063 on the colony forming ability of 11Z and EESC cells. (D) Experimental group was treated with VS-6063. After 24 hours, the migration ability was compared between the two groups. (E) Cell microscopic image of induced decaturation of HESC cells by VS-6063 in vitro. (F) Effect of VS-6063 on mRNA levels of PRL and IGFBP1 in decidualization. (G) fect of VS-6063 on PRL and IGFBP1 protein expression levels in decidualization. (The Student’s t-test was used for data analysis. All data represent mean ± SEM. *P<0.05, **P<0.01, ***P<0.001, ****P<0.0001)

**
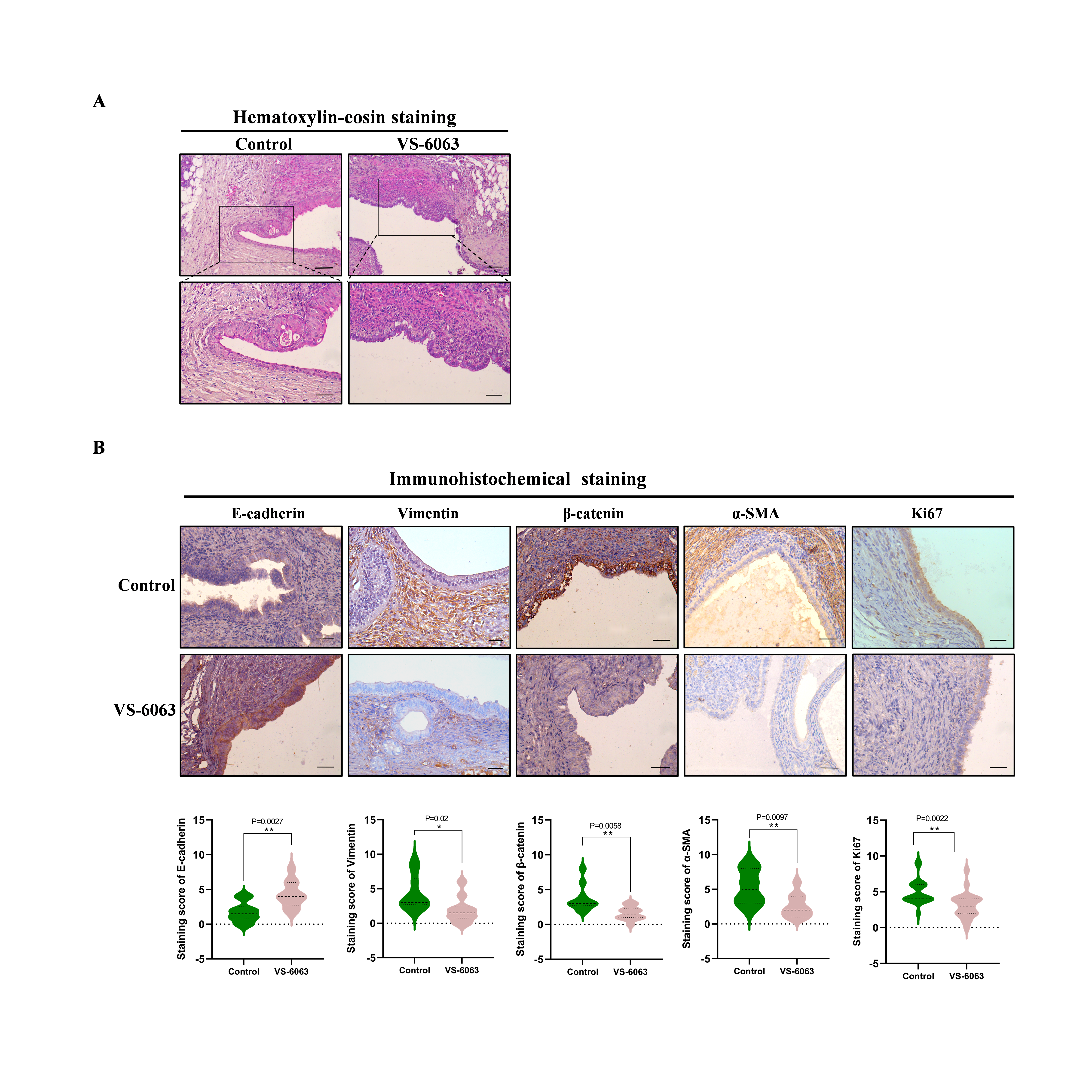
Figure S6. VS-6063 have therapeutic effects on endometriosis in vivo, related to Figure 7**

(A) Hematoxylin-eosin staining of endometriotic tissues in mice (Scale bar, 20µm). (B) Representative photographs of E-cadherin, Vimentin, β-catenin, α-SMA, Ki67 staining of endometriotic tissues in mice (Scale bar, 20µm). (The Student’s t-test was used for data analysis. All data represent mean ± SEM. *P<0.05, **P<0.01, ***P<0.001, ****P<0.0001)
